# Anti-Microbial, Thermal, Mechanical, and Gas Barrier Properties of Linear Low-Density Polyethylene Extrusion Blow-Molded Bottles

**DOI:** 10.3390/polym16131914

**Published:** 2024-07-04

**Authors:** Saleh Alkarri, Muhammed Naveed, Fatimah Alali, Jérôme Vachon, Aaron Walworth, Abigail Vanderberg

**Affiliations:** 1 School of Packaging, Michigan State University, 448 Wilson Road, East Lansing, MI 48824-1223, USA; 2 Almoosa College of Health Sciences, Ain Najm Rd, Al Mubarraz 36422, Saudi Arabia; 3 SABIC, P.O. Box 319, 6160 AH Geleen, The Netherlands; 4 Center for Advanced Microscopy, Michigan State University, 578 Wilson Road, CIPS Bldg, Rm B-6B, East Lansing, MI 48824-1223, USA

**Keywords:** anti-microbial activity, *E. coli* K-12 MG1655, anti-microbial agents, thermal embossing, extrusion blow molding

## Abstract

Microbial contamination can occur on the surfaces of blow-molded bottles, necessitating the development and application of effective anti-microbial treatments to mitigate the hazards associated with microbial growth. In this study, new methods of incorporating anti-microbial particles into linear low-density polyethylene (LLDPE) extrusion blow-molded bottles were developed. The anti-microbial particles were thermally embossed on the external surface of the bottle through two particle deposition approaches (spray and powder) over the mold cavity. The produced bottles were studied for their thermal, mechanical, gas barrier, and anti-microbial properties. Both deposition approaches indicated a significant enhancement in anti-microbial activity, as well as barrier properties, while maintaining thermal and mechanical performance. Considering both the effect of anti-microbial agents and variations in tensile bar weight and thickness, the statistical analysis of the mechanical properties showed that applying the anti-microbial agents had no significant influence on the tensile properties of the blow-molded bottles. The external fixation of the particles over the surface of the bottles would result in optimum anti-microbial activity, making it a cost-effective solution compared to conventional compounding processing.

## 1. Introduction

Extrusion blow molding is a common manufacturing method for generating hollow plastic containers such as bottles and jars. These containers are utilized in many industries, including food and beverage, pharmaceutical, cosmetic, and household product production [1]. However, microbial contamination can occur on the surfaces of blow-molded bottles, necessitating the development and application of effective anti-microbial treatments to mitigate the hazards associated with microbial growth. For instance, the shelf life of a contained sensitive material stored in blow-molding plastic containers is highly dependent upon the level of sterility of the plastic. Sterilization techniques and aseptic filling are thus commonly employed [2]. Additionally, microorganisms can be transmitted onto the surfaces of blow-molded bottles through various means [3]. For instance, an individual may touch contaminated surfaces like door handles, countertops, or packaging materials and then inadvertently transfer microbes onto the bottles through direct contact. Microorganisms can also be disseminated through air movement within a production facility. In environments where microbial aerosols are prevalent, such as hospitals or crowded spaces, airborne microorganisms can also land on the surfaces of blow-molded bottles in their end use location. Airborne fungal spores in a room have been shown to settle on bottles, leading to contamination [4].

The surfaces of extrusion blow-molded bottles provide an ideal environment for bacterial growth due to their smooth texture and organic residues [1]. Colonization of surfaces by bacteria such as *Escherichia coli* (*E. coli*), *Staphylococcus aureus* (*S. aureus*), and *Pseudomonas aeruginosa* (*P. aeruginosa*) can result in rapid bacterial growth and the development of biofilms. Kim et al. (2020) state that biofilms may cause food degradation and perhaps spread diseases to customers. Biofilms can also serve as long-term bacterial contamination reservoirs [5]. To prevent bacterial colonization, creating anti-microbial surfaces for blow-molded bottles is crucial [6]. Growing demand for plastic containers in various industries has rapidly increased the use of blow molding in recent years [7,8]. Chadha et al. (2022) remark that population growth, urbanization, and the increased demand for packaged goods are all factors that affect the extrusion blow molding industry’s market size. The extrusion blow molding market was estimated to be 100 billion USD in 2020, and according to Chadha et al. (2022), it is expected to increase at a CAGR of 5% between 2021 and 2028 [9]. Polyethylene (PE) and polyethylene terephthalate (PET) are two of the most widely used plastics in blow molding and account for a sizable share of global plastic consumption [10].

Advancements in the development of anti-microbial agents for plastics application have resulted in the emergence of two distinct categories, leachable and non-leachable agents, according to Gulati, Sharma, and Sharma (2021). Leachable agents can release anti-microbial compounds from the polymer matrix, offering sustained efficacy [11]. However, concerns about potentially harmful substance release arise. For example, silver nanoparticles embedded in the polymer gradually release silver ions when they come in contact with microorganisms, leading to the detection of silver in good it intends to protect [12]. In contrast, non-leachable agents remain fixed on the plastic surface, offering immediate and localized anti-microbial effects. Non-leachable agents include Mg(OH)_2_ and chitosan, a natural biopolymer that has shown promise in anti-microbial packaging [12]. While non-leachable agents may be susceptible to wear and degradation, recent research has focused on formulating and evaluating these agents for enhanced performance and safety [11]. Since non-leachable agents require direct contact with microbes to have an effect, they are better suited as coatings rather than compounded articles, where the additive would be dispersed inside the matrix [13].

Various anti-microbial substances have been investigated in the field of extrusion blow molding. Incorporating copper (Cu) may be a viable method for doping in Mg(OH)_2_, thereby enabling the modulation of the material’s optical bandgap. CuO can modulate electron field emission characteristics owing to its low potential barrier [14]. However, more research is required to determine its effectiveness.

Anti-microbial agents have been incorporated into the process through different methods. One of these methods is melt-compounding with plastics like PE and PET [15]. Another method is the use of coating technologies applied to the exterior of the bottles [16].

In the case of melt-compounding, the anti-microbial agents are mixed with the plastic materials during the manufacturing process, creating a uniform matrix of the polymer and additive [17]. According to Huang et al., the coating process applies a layer of anti-microbial agent to the bottle surfaces, resulting in a thin layer, with thicknesses typically ranging from a few to tens of micrometers [16]. The adjustment of concentrations and thicknesses of anti-microbial agents is contingent upon the targeted degree of anti-bacterial efficacy and the particular demands of the application, as noted by Huang et al. [16]. Notably, the types of anti-microbial agents and the coating techniques employed may exhibit variations across different investigations, contingent upon factors such as the specific microorganisms being targeted and the intended duration of anti-microbial efficacy [16].

The study conducted by Hutasoit et al. has revealed that Cu-infused Mg(OH)_2_ could exhibit robust anti-bacterial characteristics against a wide range of bacteria, including Gram-positive and Gram-negative strains, such as *Salmonella* spp., *E. coli*, and *S. aureus* because of the *Cu* and *Mg* contents [18]. Another study has shown that the alkyd resin nanocomposite derived from palm oil containing Mg(OH)_2_/MgO colloidal NPs has displayed catalytic performance and anti-microbial activity. Some bacteria, including methicillin-resistant *S. aureus* and *P. aeruginosa*, are killed by Mg(OH)_2_ and Cu(OH)_2_, respectively [19]. According to Birkett et al., the concentration and thickness of an anti-microbial coating greatly affect its efficiency [20]. Higher concentrations of anti-microbial compounds are typically associated with increased anti-microbial action. Darvish et al. pointed out that obtaining the optimum concentration is essential to avoid unintended implications, such as altering the polymer’s physical characteristics or making leaching of the agent more likely [21]. Since the integrity of extrusion blow-molded bottles must be preserved during anti-microbial treatment, it is crucial to determine the concentration of the anti-microbial agent needed to achieve this goal [22]. Similarly, the thickness of the coating layer influences the anti-microbial performance. Thicker coatings can increase protection against microbial contamination [23]. However, excessively thick coatings may be prone to cracking or peeling, compromising their effectiveness [24]. Recent publications have highlighted the importance of optimizing the coating thickness to balance both anti-microbial activity and coating durability [25,26].

Among the various anti-bacterial agents mentioned, copper-infused Mg(OH)_2_ has exhibited potential efficacy against Gram-positive and Gram-negative bacteria [27]. The broad-spectrum anti-bacterial activity of copper ions released from copper-infused Mg(OH)_2_ targets DNA, proteins, and bacterial cell membranes [27]. This mechanism makes it effective against a wide range of bacteria, including those with varying cell wall structures [6,27].

The effectiveness of anti-microbial substances such as Mg(OH)_2_, Cu(OH)_2_, MgO, CuCl_2_, and ZnO can differ, depending on the type of bacteria [28]. According to research findings, CuCl_2_ exhibits noteworthy inhibitory properties against the proliferation of Gram-negative bacterial strains such as *E. coli* and *P. aeruginosa*. In contrast, it has been reported that MgO and ZnO exhibit greater efficacy against Gram-positive bacteria [29]. The observed variation in efficacy underscores the diverse antimicrobial properties of these compounds, as reported by Jakubovskis et al. [30].

The mechanisms by which anti-microbial particles induce cell death are multifaceted and contingent upon the particular agent utilized. Some examples of anti-microbial modes of action are the disruption of cell membranes, the production of reactive oxygen species (ROS), the suppression of enzymatic activities, or the induction of damage to DNA. According to Imani et al., the anti-microbial effectiveness of Cu-infused Mg(OH)_2_, Mg(OH)_2_, Cu(OH)_2_, MgO, CuCl_2_, and ZnO is often attributed to their multi-modal actions [31]. Imani et al. report that one particular mechanism entails the interference of bacterial cell membranes through the utilization of distinct anti-microbial nanoparticles, namely Mg(OH)_2_, Cu(OH)_2_, MgO, CuCl_2_, and ZnO [31]. The NPs can interact with the bacterial cell membrane, thereby compromising its structural integrity and the consequent release of its cellular constituents. The disruption of the membrane structure and function results in the disturbance of crucial cellular processes and eventual cell death, as reported by Imani et al. [31].

An additional mechanism involves the production of ROS. According to Smaoui et al., specific anti-microbial particles, including Mg(OH)_2_ and Cu-infused Mg(OH)_2_, can produce ROS upon exposure to moisture or light. ROS, such as hydroxyl radicals and superoxide ions, elicit oxidative harm within bacterial cells, thus deactivating them [32].

The suppression of enzymatic activity represents another pivotal mechanism utilized by certain anti-microbial particles. Peters et al. have demonstrated the effectiveness of Cu-infused Mg(OH)_2_ and CuCl_2_ in impeding the function of crucial enzymes in bacterial cells [33]. This interference with enzymatic function leads to the impairment of crucial metabolic processes, ultimately culminating in the demise of the bacteria [33].

In addition, it has been observed that anti-microbial agents containing copper, such as Cu-infused Mg(OH)_2_ and Cu(OH)_2_, can potentially induce DNA damage in bacterial cells. According to Rojas et al., the agents interact with bacterial DNA, resulting in structural harm and disruption of its replication and transcription mechanisms [34]. The amalgamation of physical and chemical mechanisms in these particles effectively contributes to their anti-microbial properties, thereby enabling them to either inhibit bacterial growth or cause bacterial death [34].

According to Gumienna et al., the regulatory approval status of anti-microbial agents utilized in blow-molding applications may differ, based on the particular agent and its intended application, as determined by the Food and Drug Administration (FDA) [35]. The authors state that certain anti-microbial agents utilized in blow molding have not obtained approval from the FDA. Some anti-microbial agents, including copper and zinc compounds, have been generally recognized as safe (GRAS) by the FDA for diverse applications [36]. The substances that have been designated as GRAS have been deemed to meet the safety requirements set forth by the FDA and are therefore suitable for use in contact with pharmaceutical or food items, as per the findings of Mania et al. [36]. Evaluating anti-microbial agents’ toxicity is critical due to its potential impact on human cells. The toxicity of various NPs, including Cu-infused Mg(OH)_2_, Mg(OH)_2_, Cu(OH)_2_, MgO, CuCl_2_, and ZnO, has been investigated in human cells through research conducted by Naz et al. [37] Their results indicate that nanoparticles typically demonstrate negligible cytotoxicity at the lower concentrations that fall within the anti-microbial range, and that they are well received by human cells [37]. However, high concentrations or prolonged exposure to specific anti-microbial agents may lead to adverse effects [38].

Furthermore, the durability and longevity of the anti-microbial effects are important aspects related to anti-microbial techniques in extrusion blow molding applications [22]. The influence of environmental conditions on the performance of anti-microbial coatings, as well as the development of sustainable and environmentally friendly anti-microbial agents, are also significant. These aspects are critical for the practical implementation and commercial viability of anti-microbial solutions in the extrusion blow molding industry [39].

Developing effective anti-microbial techniques in blow molding applications is crucial to ensuring product safety and effective protection from microbial contamination. The utilization of anti-microbial agents, including Cu-infused Mg(OH)_2_, Mg(OH)_2_, Cu(OH)_2_, MgO, CuCl_2_, and ZnO, has shown promising results in inhibiting bacterial growth on blow-molded bottle surfaces. These agents’ concentrations, thicknesses, and mechanisms of action play essential roles in their anti-microbial efficacy. Comprehensive toxicity evaluations are necessary in the future to ensure the safety of these agents for human health.

## 2. Experimental

### 2.1. Materials

Linear low-density polyethylene (LLDPE) copolymer (DOWLEX 2045G grade) was obtained in pellet form from Dow Chemical Company (Midland, MI, USA). These pellets have the following characteristics: melting point = 120.0 °C, density = 920 kg/m^3^, and melt flow index (MFI) = 1 g/10 min (190 °C/2.16 kg). Mg(OH)_2_, Cu-infused Mg(OH)_2_, MgO, and Cu(OH)_2_ (purity: 99.99%) were gifted by Aqua Resources (Fort Walton Beach, FL, USA), both as dry powder and slurry (dispersed in water). ZnO (purity: 99.00%) was obtained from American Elements (Los Angeles, CA, USA) as a slurry (dispersed in water). Isopropyl alcohol (purity: 99.99%) was obtained from Macron Fine Chemicals (Center Valley, PA, USA).

### 2.2. Preparation of Anti-Microbial Suspensions for Internal Mold Cavity Spray

**Figure 1:**
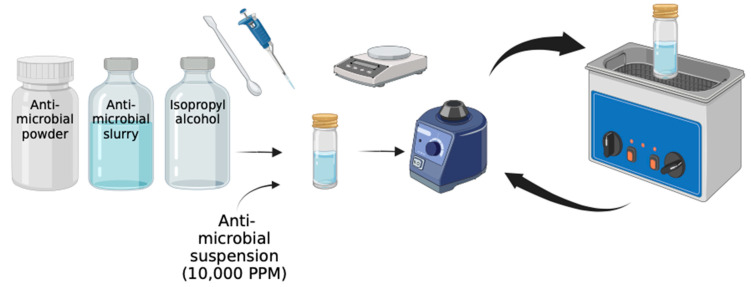
Preparation of the anti-microbial suspensions. Reprinted from ref. [26].

The Mg(OH)_2_ NPs were obtained commercially in slurry form (7 wt.% Mg(OH)_2_ and 93 wt.% water). The Mg(OH)_2_ slurry (14.3 mL) was diluted with isopropyl alcohol (85.7 mL) to prepare an Mg(OH)_2_ suspension at a concentration of 10 mg/mL. The Cu-infused Mg(OH)_2_ NPs were obtained commercially as a slurry (7.47 wt.% Cu-infused Mg(OH)_2_ and 92.53 wt.% water). The Cu-infused Mg(OH)_2_ slurry (1.34 mL) was diluted with isopropyl alcohol (8.66 mL) to prepare a Cu-infused Mg(OH)_2_ suspension at a concentration of 10 mg/mL. The MgO NPs (500 mg) were combined with isopropyl alcohol (50 mL) to prepare an MgO NPs suspension at a concentration of 10 mg/mL. The Cu(OH)_2_ NPs were obtained commercially as a slurry (22.25 wt.% Cu(OH)_2_ and 77.75 wt.% water). The Cu(OH)_2_ slurry (1.75 mL) was diluted with isopropyl alcohol (48.25 mL) to prepare a Cu(OH)_2_ NPs suspension at a concentration of 10 mg/mL. The ZnO NPs were obtained commercially as a slurry (20 wt.% ZnO and 80 wt.% water). The ZnO slurry (2 mL) was diluted with isopropyl alcohol (48 mL) to prepare a ZnO NPs suspension at a concentration of 10 mg/mL. The NP suspensions were vortexed at maximum speed for 30 s, and subsequently sonicated in an ultrasonic bath (Branson 2510 Ultrasonic Sonicator, Commack, NY, USA) at 23 °C for 10 min to ensure that the NPs were uniformly dispersed. After sonication, the suspension was vortexed once more at maximum speed for 30 s (Figure 1) [26].

### 2.3. Blow Molding

**Figure 2:**
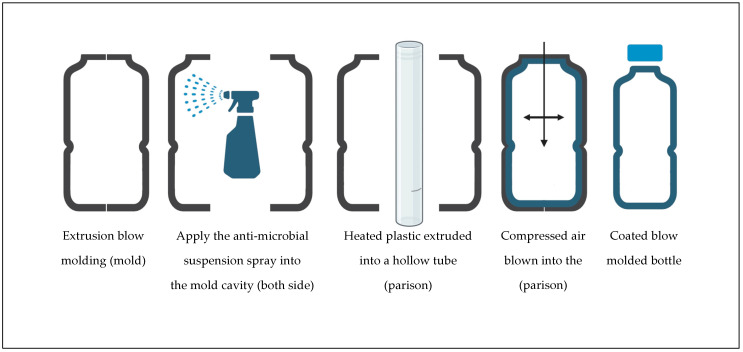
Illustration of extrusion blow molding.

The bottles were produced using a Bekum H111S extrusion blow molder (Serial 974948-5-056), outfitted with a chilled single cavity aluminum mold [40].The mold chiller was type BMB-II-B, manufactured by Fasti USA (Elgin, IL, USA). The mold was a 500 mL round bullet/cosmo-style bottle mold (please see Section S1 in the Supplementary Information (SI) Document). The blow molder was warmed up for at least one hour before each processing run. The internal cavity of the mold was cleaned with 100% isopropyl alcohol and non-woven polypropylene (PP) fabric, followed by compressed air, after each cycle when the anti-microbial suspension was applied. The first five containers retrieved from the machine were discarded as a method of purging the machine. Then five neat LLDPE bottles were produced, removed in order, and placed inverted (finish down) in a divided, numbered sample tray. For anti-microbial treatment, both sides of the mold cavity were sprayed evenly with the anti-microbial suspension five times (equivalent to approximately 1.8 mL for each cavity side) using 30 mL capacity fine mist spray bottles (Anyumocz brand, CN) and left to dry for 50 s before each cycle began (Figure 2).

The Mg(OH)_2_ nanoplatelets were applied to the mold cavity as a powder using a cosmetic embossing powder tool (brand: BAOFALI) before the production cycle was initiated (please see Section S2 in the SI document).

**Figure 3:**
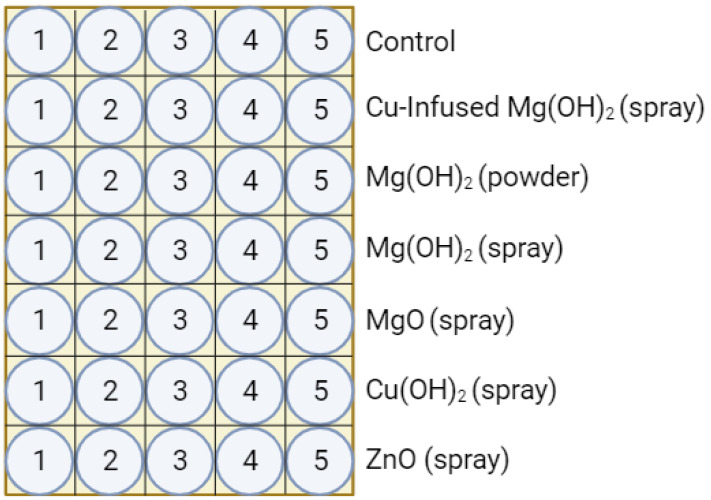
Sample tray layout.

The containers were stored inverted in divided trays to give them time to cool and to prevent the flash from becoming fused to the other containers. A total of 30 treated samples, plus 5 controls, were made per run. The bottles were laid out in the sample trays, as shown in Figure 3. Five minutes after production was complete, the flash was removed manually by twisting. The containers were then labeled by tray location and placed right-side up in a new sample tray. The sample tray was labeled with the date, run number, and manufacturing method.

### 2.4. Sample Preparation of Extrusion Blow Molding Bottles

#### 2.4.1. Extrusion Blow-Molded Bottle Washing Techniques

**Figure 4:**
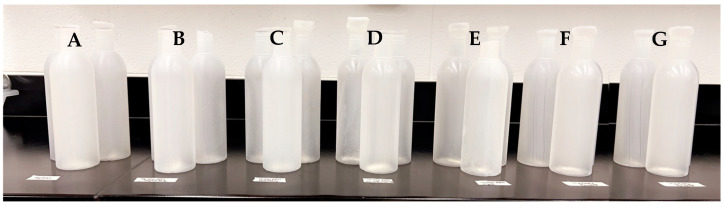
Various LLDPE extrusion blow-molded bottles; neat LLDPE (**A**) and thermally embossed LLDPE with Cu-infused Mg(OH)_2_ spray (**B**), Mg(OH)_2_ powder (**C**), Mg(OH)_2_ spray (**D**), MgO spray (**E**), Cu(OH)_2_ spray (**F**), and ZnO spray (**G**).

Each set of extrusion blow-molded bottles (Figure 4) was dipped in a plastic container filled with a deionized water (diH_2_O), and the outer surfaces were rubbed with a nitrile-gloved hand to assure the planned characterizations would show only the affixed particles that were adhered to the surfaces. The plastic container was washed and refilled with diH_2_O after each set of treatments. After washing, the bottles were placed vertically on a piece of aluminum foil to dry for at least 48 h at room temperature.

#### 2.4.2. Sample Preparation of the Extruded Blow-Molded Bottles

The extrusion blow-molded bottles were cut down to different shapes and sizes as follows: (i) circular disk (dimension: 20 mm × 0.48 mm), used for anti-microbial, SEM, and EDX; (ii) square shape (dimension: 50 mm × 50 mm × 0.48 mm), used for barrier studies; and (iii) tensile bars (dimension: 127 mm × 25.4 mm × 0.48 mm), used to test the mechanical properties. A JDC PRECISION SAMPLE CUTTER (Thwing-Albert Instrument Company, Philadelphia, PA, USA, Model: JDC 1-10, Serial No 36757) was used to cut the strips of sheets into one inch widths.

### 2.5. Experimental Design and Statistical Analysis

The means, standard deviations, and percentage changes obtained from the investigation of various properties in the LLDPE extrusion blow-molded bottles thermally embossed with various anti-microbial nanoparticles were evaluated and compared using JMP software (JMP Pro 16.1.0 (539038), SAS Institute Inc., Cary, NC, USA). All experiments were independently replicated at least three times to properly evaluate the properties of the LLDPE extrusion blow-molded bottles.

### 2.6. Characterization

#### 2.6.1. Scanning Electron Microscopy (SEM) and Energy Dispersive X-ray Spectroscopy (EDX)

SEM was used to characterize unprocessed anti-microbial nanoparticles and disks of blow-molded bottles thermally embossed with anti-microbial agents. The samples were imaged using a JEOL 7500F field emission SEM (JEOL Ltd., Tokyo, Japan). EDX was performed using an Oxford Instruments AZtec system (Oxford Instruments, High Wycomb, Bucks, UK) attached to the SEM. Prior to SEM and EDX analysis, dry powders were adhered to aluminum stubs using high vacuum carbon tabs (SPI Supplies, West Chester, PA, USA). Slurry suspensions of nanoparticles were dried by placing two drops of solution onto silicon wafers (Ted Pella, Inc., Redding, CA, USA), and then the wafers were attached to the stubs. The disks of blow-molded bottles were attached to the stubs using epoxy glue (System Three Resins, Inc., Aubur, WA, USA). The samples were coated with either iridium or osmium. Iridium coating was performed in a Quorum Technologies/Electron Microscopy Sciences Q150T sputter coater (Quorum Technologies, Laughton, East Sussex, UK). A Tennant20 CVD coater (Meiwafosis Co., Ltd., Osaka, Japan) was used for osmium coating.

#### 2.6.2. Differential Scanning Calorimetry (DSC)

The TA Instrument, Model Q100 system, used DSC to determine the crystallization temperatures and melting points of the LLDPE samples. All samples were analyzed in triplicate. The temperature range for analysis was between −20 to 250 °C. The rate of temperature change was 10 °C min^−1^. The process was then paused at ^−1^. The samples were then cooled at a rate of 10 °C min^−1^ to −20 °C and then re-heated at the same rate to 250 °C. All thermal responses were recorded to determine the crystallization temperature and melting point. The heat of fusion values were used to calculate the crystallinity degrees of the LLDPE samples. These values were determined from the second heating runs and evaluated according to Equation (1):

(1) $$X_{c}\left(\%\right)=\left[\frac{∆H_{c}}{∆H_{0}\cdot W}\right]\times 100$$

where *X_c_* is the crystallinity of the LLDPE samples, $∆H_{0}$ is 100% crystalline LLDPE enthalpy of fusion [279 J/g] [41], $∆H_{c}$ is the heat of fusion, and *W* is the LLDPE fraction in the composite (weight).

#### 2.6.3. Thermogravimetric Analysis (TGA)

The heat resistance and thermal decomposition of the LLDPE samples were evaluated using a Q-50 Thermogravimetric Analyzer (TGA) (TA Instruments, New Castle, DE, USA). Each sample, weighing between 6–10 mg, was subjected to heating at a rate of 10 °C/min up to a maximum of 600 °C under a nitrogen purge of 60 mL/min. A minimum of three replications was conducted for every sample, and the average result of the TGA data was used for analysis.

#### 2.6.4. Tensile Properties

The test specimens (five replicates) were maintained at standard lab conditions (23 °C, 50% RH) for a minimum period of 40 h prior to testing for tensile measurements. Following the standards of ASTM D882-18 (Standard Test Method for Tensile Properties of Thin Plastic Sheeting), the tensile tests were conducted using an Instron tensile testing system (model 5565, Minneapolis, MA, USA). The specimens were held by pneumatic grips with an initial grip separation of 33 mm. The extension was controlled at a constant speed of 500 mm/min. The test concluded when the sample broke or when a decrease of 60% of peak force was reached.

#### 2.6.5. Barrier Properties

##### Water Vapor Transition Rate (WVTR)

The determination of WVTR was carried out at 37.8 °C and 90% relative humidity using a Permatran-W system (Model 3/34, Mocon Inc., Minneapolis, MN, USA), adhering to the ASTM F1249 standards. The calculation of water vapor permeation was achieved by multiplying the thickness of the film specimen (two duplicates of each LLDPE sample) by the observed values for water vapor transmission. Specimens were masked using self-adhesive foil to provide an exposed surface area of 12.57 cm^2^.

##### Oxygen Transition Rate (OTR)

The OTR of the LLDPE film samples was determined at 23 °C and 50% relative humidity using an Ox-Tran system (model 2/22, Mocon Inc., Minneapolis, MN, USA), in accordance with ASTM D3985. This process involved testing two duplicates of each LLDPE sample. Specimens were masked using self-adhesive foil to provide an exposed surface area of 12.57 cm^2^. The test gas was 100% O_2_.

#### 2.6.6. Anti-Microbial Testing Method

The anti-microbial activities of the disks were evaluated sequentially for all experiments using the *E. coli* K-12 MG165. The stock culture was stored at −80 °C in a ThermoFisher TSX400 system. A streak containing the *E. coli* K-12 MG165 from the stock culture was then applied onto Tryptic Soy Agar (TSA) plates. A single colony was selected after a 24 h incubation at 37 °C and transferred to 5 mL of Tryptic Soy Broth (TSB). The broth was incubated for 18 h at 37 °C. After incubation, the cultures were centrifuged using a Fisher Scientific accuSpin micro 17 R centrifuge at 13,000× *g*. A total of 1 mL of culture was centrifuged for 5 min, and the supernatant was discarded. The cells were suspended in 1 mL of phosphate-buffered saline (PBS, Crystalgen, Innovation for Science, Commack, NY, USA), by vortexing. A total of 11.5 mL of PBS was added to the cell suspension after transferring it to a 15 mL tube.

**Figure 5:**
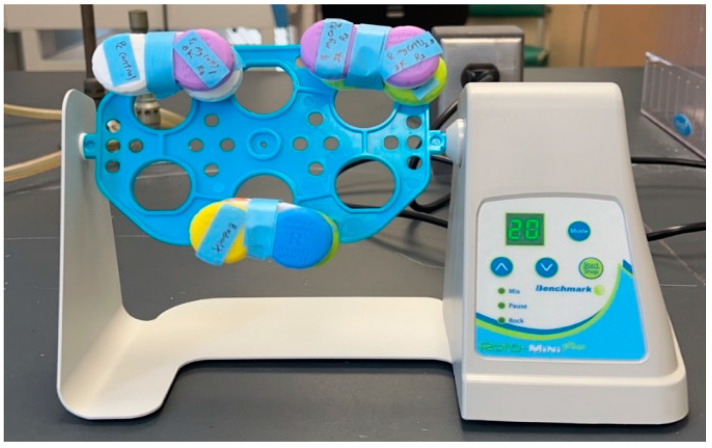
The pods were attached with tape to the mini rotator device for consistent agitation of the bacterial broth and surface renewal of the disks inside the pods. The blue tray holding the pods rotates around the horizontal axis. Reprinted from Ref. [26].

Aliquots of this suspension were then exposed to the different types of disks. Every disk was placed separately in a pod using a contact lens cases manufactured by Bosch + Lomb. For each pod containing a single disk, 1 mL of bacterial suspension was added to submerge the disk into the culture broth; subsequently, the pods were closed. The pods were then attached to a mini rotator (Benchmark Scientific, Roto Mini Plus R 2024, Sayreville, NJ, USA) and rotated at 20 rpm (Figure 5) around the machine’s horizontal axis to continuously agitate the broth and cause liquid renewal on the surface of the disks [26]. At 0, 4, and 24 h intervals, a 100 mL sample of the bacterial suspension was removed for the appropriate number of 1:10 dilutions, then incubated at 37 °C overnight after being plated on TSA. The bacterial cell density at each time point was determined by enumerating the colony-forming units (CFU) using a Scan300 (InterScience, Saint-Nom-la-Bretèche, France). Neat LLDPE disks were used as a “negative” control sample, while metallic copper disks were used as a “positive” control sample. In addition, the anti-microbial activity of different LLDPE composite disks was tested individually.

## 3. Results

### 3.1. Scanning Electron Microscopy (SEM) and Energy Dispersive X-ray Spectroscopy (EDX)

**Figure 6:**
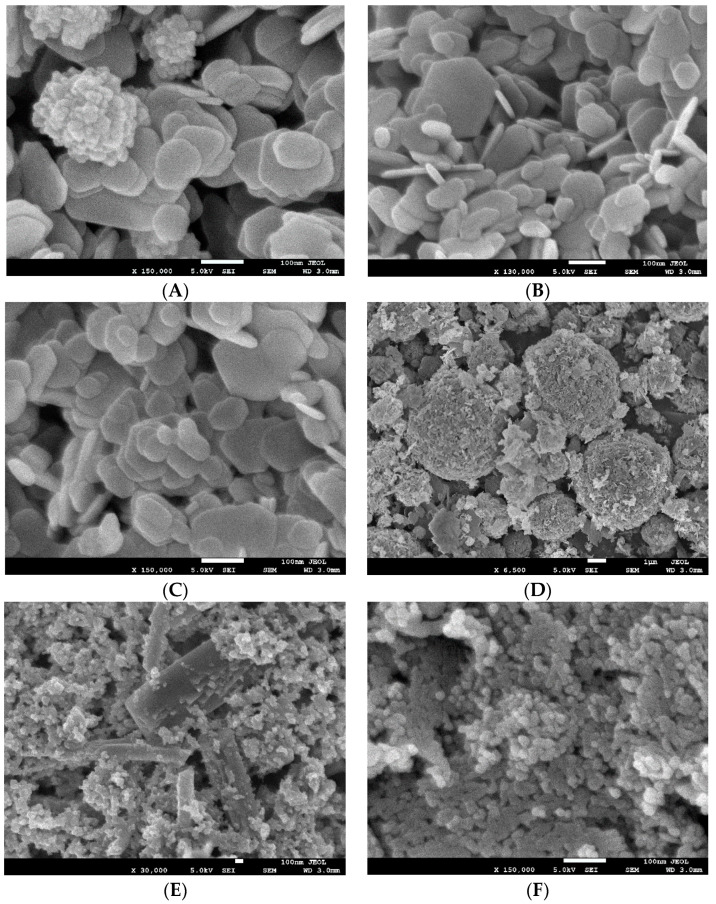
The SEM images of various pure anti-microbial NPs; Cu-infused Mg(OH)_2_ spray (**A**), Mg(OH)_2_ powder (**B**), Mg(OH)_2_ spray (**C**), MgO spray (**D**), Cu(OH)_2_ spray (**E**), and ZnO spray (**F**).

The SEM characterization for the various pure anti-microbial NPs is presented in Figure 6.

**Figure 7:**
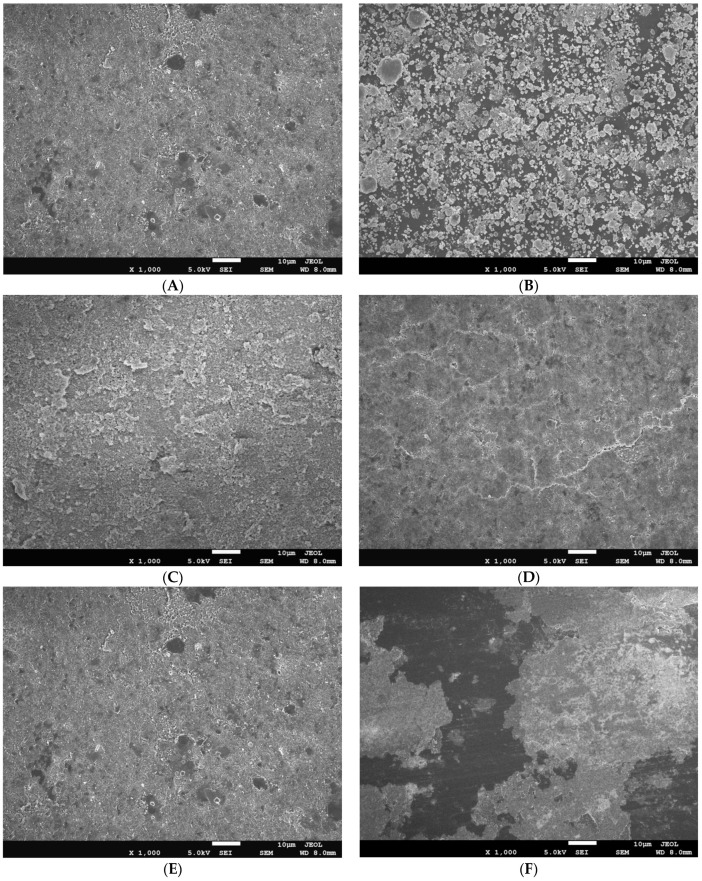
The SEM images of blow-molded bottles thermally embossed with Cu-infused Mg(OH)_2_ spray (**A**), Mg(OH)_2_ dry powder (**B**), Mg(OH)_2_ spray (**C**), MgO spray (**D**), Cu(OH)_2_ spray (**E**), and ZnO spray (**F**).

The SEM characterization of the studied extrusion blow-molded LLDPE bottles thermally embossed with variety of anti-microbial NPs is presented in Figure 7.

**Figure 8:**
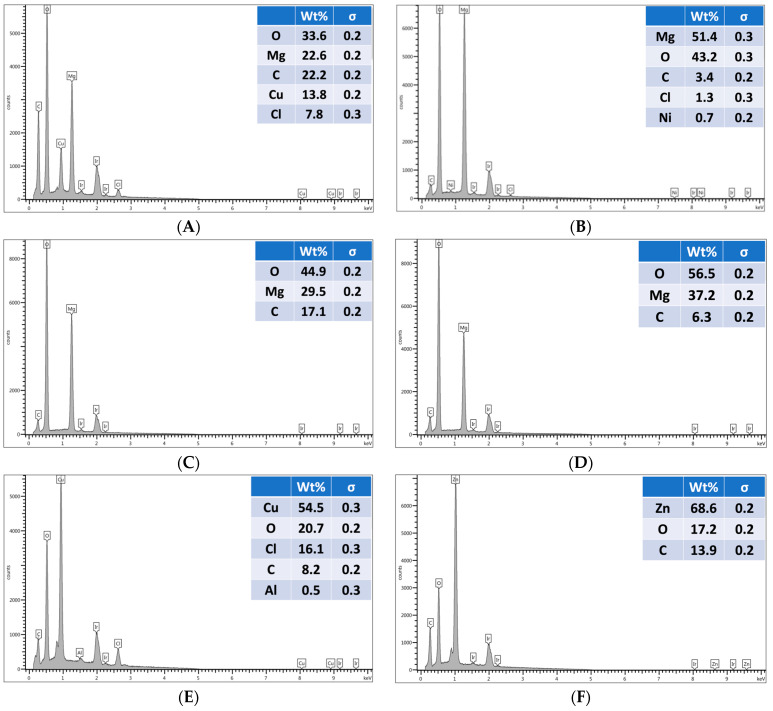
The EDX spectrums for blow-molded bottles thermally embossed with Cu-infused Mg(OH)_2_ spray (**A**), Mg(OH)_2_ dry powder (**B**), Mg(OH)_2_ spray (**C**), MgO spray (**D**), Cu(OH)_2_ spray (**E**), and ZnO spray (**F**).

The EDX characterization for the studied extrusion blow-molded LLDPE bottles thermally embossed with variety of anti-microbial NPs is presented in Figure 8. To review a detailed report of the EDX mapping, please see Section S3 in the SI document.

### 3.2. Differential Scanning Calorimetry (DSC)

**Figure 9:**
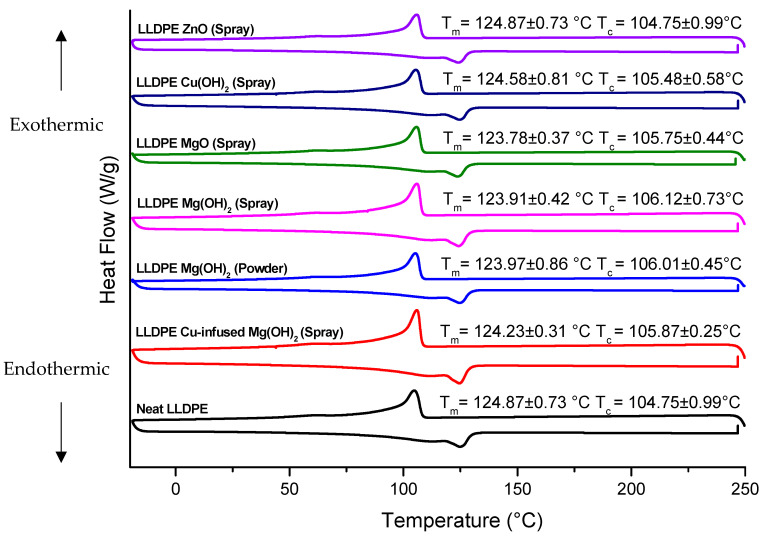
The thermal cycle of various extrusion blow-molded LLDPE bottles obtained from the DSC data.

The thermal behaviors of the studied extrusion blow-molded LLDPE bottles thermally embossed with variety of anti-microbial NPs are presented in Figure 9, and the summarized values along with the crystallinity (%) are listed in Table 1.

### 3.3. Thermogravimetric Analysis (TGA)

**Figure 10:**
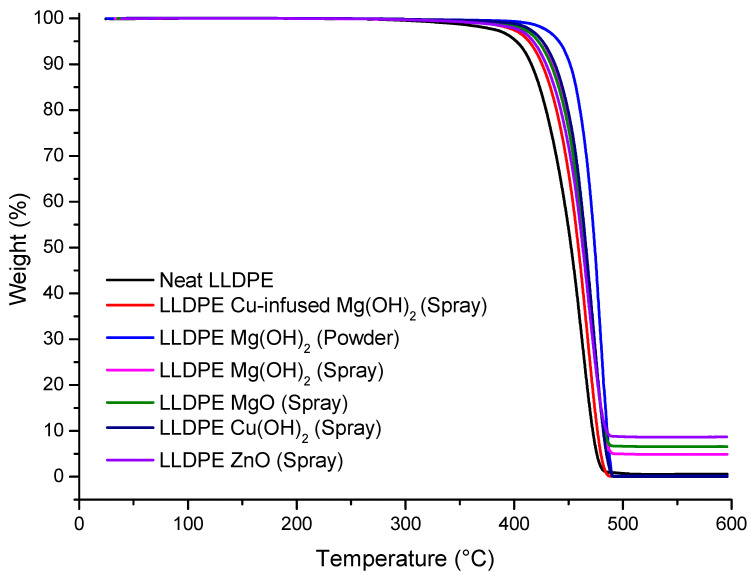
The thermal decomposition properties of various extrusion blow-molded LLDPE bottles obtained from the TGA data.

The thermal stability of each of the studied extrusion blow-molded LLDPE bottles thermally embossed with variety of anti-microbial NPs is presented in Figure 10, and the summarized values of the temperatures at which 5% weight loss occurred and the estimated residual (wt.%) at 600 (°C) are listed in Table 2.

### 3.4. Tensile Properties

**Figure 11:**
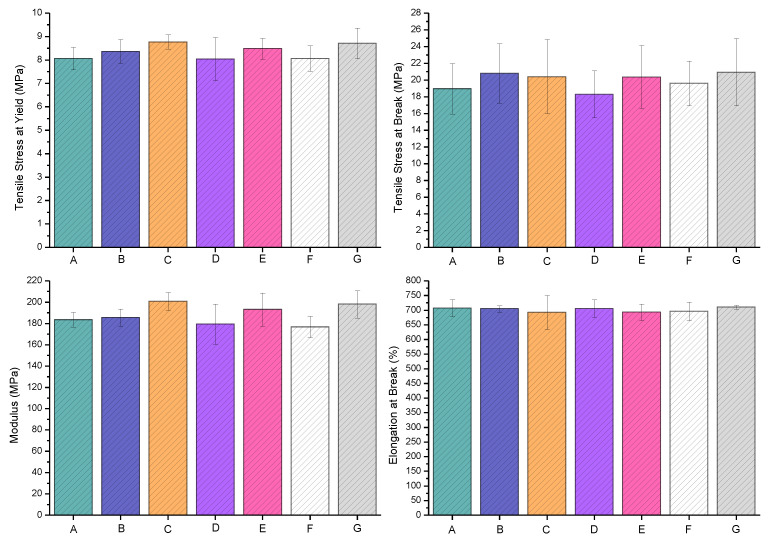
Tensile properties of various extrusion blow-molded bottles: Neat LLDPE (A), LLDPE Cu-infused Mg(OH)_2_ spray (B), LLDPE Mg(OH)_2_ powder (C), LLDPE Mg(OH)_2_ spray (D), LLDPE MgO spray (E), LLDPE Cu(OH)_2_ spray (F), and LLDPE ZnO spray (G).

The tensile properties of various extrusion blow-molded bottles thermally embossed with variety of anti-microbial NPs are presented in Figure 11.

### 3.5. Barrier Properties

**Figure 12:**
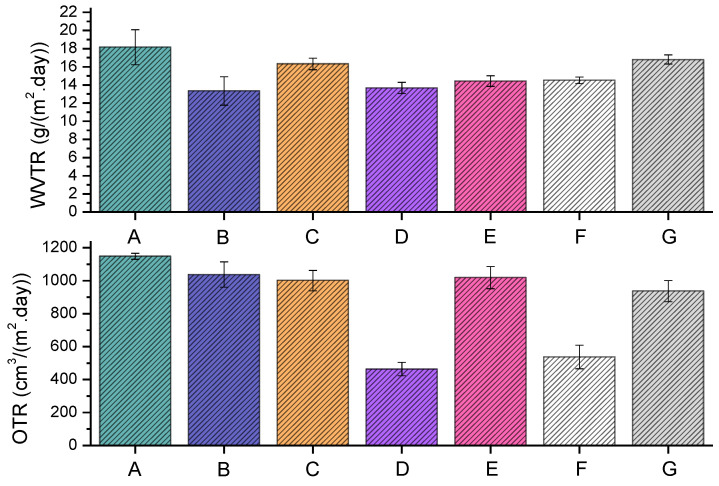
The WVTR and OTR of each graph represent the following samples on the x-axis; neat LLDPE (A), LLDPE Cu-infused Mg(OH)_2_ spray (B), LLDPE Mg(OH)_2_ powder (C), LLDPE Mg(OH)_2_ spray (D), LLDPE MgO spray (E), LLDPE Cu(OH)_2_ spray (F), and LLDPE ZnO spray (G).

The barrier properties of various extrusion blow-molded bottles thermally embossed with variety of anti-microbial NPs are presented in Figure 12.

### 3.6. Anti-Microbial Properties

**Figure 13:**
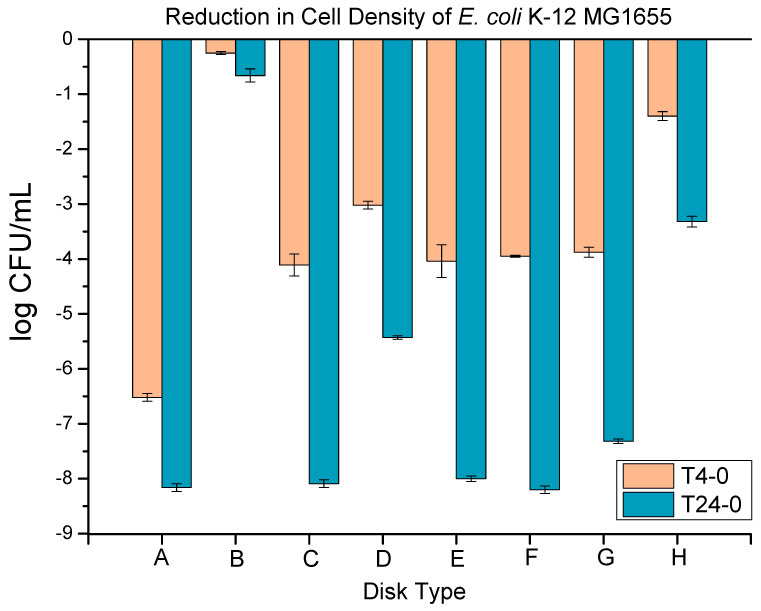
The anti-microbial data obtained for the extrusion blow-molded bottles thermally embossed with several types of anti-microbial agents at 4 and 24 h. Metallic copper—positive control (A), Neat LLDPE—negative control (B), LLDPE Cu-infused Mg(OH)_2_ spray (C), LLDPE Mg(OH)_2_ powder (D), LLDPE Mg(OH)_2_ spray (E), LLDPE MgO spray (F), LLDPE Cu(OH)_2_ spray (G), and LLDPE ZnO spray (H).

The anti-microbial properties of various extrusion blow-molded bottles thermally embossed with variety of anti-microbial NPs are presented in Figure 13.

## 4. Discussion

SEM was used to characterize nanoparticle size and morphology, as shown in Figure 6. Mg(OH)_2_ nanoparticles appeared as platelets ranging in size from 50–150 nm. Cu-infused Mg(OH)_2_ contained similar platelets of Mg(OH)_2_, with the addition of spherical, lobed Cu nanoparticles approximately 150 nm in diameter. MgO nanoparticles formed spherical agglomerates comprised of angular flakes. The flake size ranged from 50–200 nm; spherical agglomerates of MgO flakes measured 1–6 µm in diameter. Cu(OH)_2_ appeared in two distinct sizes; large rectangular crystals greater than 500 nm in length were mixed with small rectangular nanoparticles of 20–50 nm in size. ZnO nanoparticles had a rounded shape measuring approximately 10–25 nm.

Blow-molded bottles thermally embossed with anti-microbial agents were analyzed using **SEM** and **EDX** to determine the uniformity of the coatings and to confirm the chemical composition of the nanoparticle layer. SEM images show uniform nanoparticle coverage in spray applied coatings of Cu-infused Mg(OH)_2_, Mg(OH)_2_, MgO, and Cu(OH)_2_. However, ZnO spray provided uneven coating, and large areas of exposed bottle surface were visible. Mg(OH)_2_ applied in powder form caused significant aggregation of nanoparticles, resulting in a spattered, irregular coating, with the bottle surface exposed throughout the disk, as shown in Figure 7. EDX analysis of uniformly coated areas of blow-molded bottles confirmed the presence and composition of the expected nanoparticles on the bottle surface, as shown in Figure 8. In a previous study, we showed that anti-microbial nanoparticles such as Mg(OH)_2_ and CuCl_2_ can be affixed to thermoplastic sheets through a thermal embossing process [42]. The Mg(OH)_2_ and CuCl_2_ nano crystals were coated on the sheet from a nano crystal suspension, dried, and then the coated sheet was heat pressed. The sheet successfully killed microbes, and the crystals that were thermally fixed on the surface were not affected by wiping or washing the surface [42]. A limitation of this approach is that such thermal embossing methods can only be applied to sheet substrates, and not to articles with complex shapes, as the heat pressing steps required to fix the crystals to the plastic’s surface are difficult to achieve for non-flat shapes.

The DSC analysis of the LLDPE extrusion blow-molded bottles thermally embossed with Cu-infused Mg(OH)_2_ (spray) showed the following **thermal properties**: a *T_m_* of 124.23 °C ± 0.10, which was 0.51% lower compared to that of the neat LLDPE bottle (124.87 °C ± 0.54), as shown in Figure 9 and Table 1; a *T_c_* of (105.87 °C ± 0.87), which was 1.07% higher compared to that of the neat LLDPE bottle (104.75 °C ± 1.32), as shown in Figure 9 and Table 1; a crystallinity (%) of 34.82% ± 1.77, which was 5.92% lower compared to that of the neat LLDPE bottle (37.01% ± 1.25), as shown in Table 1; the temperature at which 5% weight loss occurred was 413.59 °C ± 0.86, which was 2.88% higher than that of the neat LLDPE (402.03 °C ± 1.62), as shown in Figure 10 and Table 2, and the estimated inorganic residual (wt.%) at around 600 (°C) was 0.00 ± 0.43, which was 100% lower than that of the neat LLDPE (0.61 ± 0.11), as shown in Figure 10 and Table 2. Overall, the anti-microbial NPs did not significantly impact the thermal properties of the LLDPE extrusion blow-molded bottles. Cu-infused Mg(OH)_2_ is a novel anti-microbial agent that had not been previously used for polymer application [43].

The LLDPE extrusion blow-molded bottles thermally embossed with LLDPE Mg(OH)_2_ (powder) showed the following **thermal properties**: a *T_m_* of 123.97 °C ± 1.54, which was 0.72% lower compared to that of the neat LLDPE bottle (124.87 °C ± 0.54), as shown in Figure 9 and Table 1; a *T_c_* of 106.01 °C ± 1.44, which was 1.20% higher compared to that of the neat LLDPE bottle (104.75 °C ± 1.32), as shown in Figure 9 and Table 1, a crystallinity (%) of 35.34% ± 0.87, which was 4.51% lower compared to that of the neat LLDPE bottle (37.01% ± 1.25), as shown in Table 1; the temperature at which 5% weight loss occurred was 441.28 °C ± 1.98, which was 9.76% higher than that of the neat LLDPE (402.03 °C ± 1.62), as shown in Figure 10 and Table 2, and the estimated inorganic residual (wt.%) occurred at around 600 (°C) (0.12 ± 0.82), which was 80% lower than that of the neat LLDPE (0.61 ± 0.11), as shown in Figure 10 and Table 2. Overall, the anti-microbial NPs did not significantly impact the thermal properties of the LLDPE extrusion blow-molded bottles. The Mg(OH)_2_ is a novel anti-microbial agent because of its unique nanoparticle sizes and shapes [44]. Mg(OH)_2_ NPs are broad spectrum anti-microbial agents. Dong et al. demonstrated the anti-microbial activity of Mg(OH)_2_ NPs against *E. coli* and the plant-associated bacterium *Burkholderia phytofirmans* [45]. Additional plant-associated pathogens, *Xanthomonas alfalfa* and *Pseudomonas syringae* (Huang et al.), were eliminated by Mg(OH)_2_ NPs, as were the oral, caries-associated bacteria *Streptococcus mutans* [46] and *Streptococcus sobrinus* (Okamoto et al.) [47]. Additional work from our laboratories showed the Mg(OH)_2_ and Copper Oxide NPs to be similar in their effectiveness against *E. coli* (Dong et al.).

The LLDPE extrusion blow-molded bottles thermally embossed with LLDPE Mg(OH)_2_ (spray) showed the following **thermal properties**: a *T_m_* of 123.91 °C ± 0.76, which was 0.77% lower compared to that of the neat LLDPE bottle (124.87 °C ± 0.54), as shown in Figure 9 and Table 1; a *T_c_* of 106.12 °C ± 0.45, which was 1.31% higher compared to that of the neat LLDPE bottle (104.75 °C ± 1.32), as shown in Figure 9 and Table 1; a crystallinity (%) of 38.41% ± 0.93, which was 3.78% higher compared to that of the neat LLDPE bottle (37.01% ± 1.25), as shown in Table 1; the temperature at which 5% weight loss occurred was 426.01 °C ± 2.32, which was 5.96% higher than that of the neat LLDPE (402.03 °C ± 1.62), as shown in Figure 10 and Table 2; and the estimated inorganic residual (wt.%) at around 600 (°C) was 4.91 ± 0.08, which was 705% higher than that of the neat LLDPE (0.61 ± 0.11), as shown in Figure 10 and Table 2. Overall, the anti-microbial NPs did not significantly impact the thermal properties of the LLDPE extrusion blow-molded bottles.

The LLDPE extrusion blow-molded bottles thermally embossed with LLDPE MgO (spray) showed the following **thermal properties**: a *T_m_* of 123.78 °C ± 0.37, which was 0.87% lower compared to that of the neat LLDPE bottle (124.87 °C ± 0.54), as shown in Figure 9 and Table 1; a *T_c_* of 105.75 °C ± 0.44, which was 0.95% higher compared to that of the neat LLDPE bottle (104.75 °C ± 1.32), as shown in Figure 9 and Table 1; a crystallinity (%) of 35.64% ± 1.34, which was 3.70% higher compared to that of the neat LLDPE bottle (37.01% ± 1.25), as shown in Table 1; the temperature at which 5% weight loss occurred was 422.12 °C ± 1.11, which was 5.00% higher than that of the neat LLDPE (402.03 °C ± 1.62), as shown in Figure 10 and Table 2; and the estimated inorganic residual (wt.%) at around 600 (°C) was 6.56 ± 0.45, which was 975% higher than that of the neat LLDPE (0.61 ± 0.11), as shown in Figure 10 and Table 2. Overall, the anti-microbial NPs did not significantly impact the thermal properties of the LLDPE extrusion blow-molded bottles. Alwaan et al. showed that the crystallinity of the blends of mLLDPE compounded with MgO was continuously increased by the loading of MgO when compared with the neat material [48].

The LLDPE extrusion blow-molded bottles thermally embossed with LLDPE Cu(OH)_2_ (spray) showed the following **thermal properties**: a *T_m_* of 124.58 °C ± 0.37, which was 0.23% lower compared to that of the neat LLDPE bottle (124.87 °C ± 0.54), as shown in Figure 9 and Table 1; a *T_c_* of 105.48 °C ± 1.61, which was 0.70% higher compared to that of the neat LLDPE bottle (104.75 °C ± 1.32), as shown in Figure 9 and Table 1; a crystallinity (%) of 40.79% ± 1.21, which was 10.21% higher compared to that of the neat LLDPE bottle (37.01% ± 1.25), as shown in Table 1; the temperature at which 5% weight loss occurred was 425.96 °C ± 0.95, which was 5.95% higher than that of the neat LLDPE (402.03 °C ± 1.62), as shown in Figure 10 and Table 2; and the estimated inorganic residual (wt.%) at around 600 (°C) was 0.03 ± 1.22, which was 95% lower than that of the neat LLDPE (0.61 ± 0.11), as shown in Figure 10 and Table 2. Overall, the anti-microbial NPs did not significantly impact the thermal properties of the LLDPE extrusion blow-molded bottles.

The LLDPE extrusion blow-molded bottles thermally embossed with LLDPE ZnO (spray) showed the following **thermal properties**: a *T_m_* of 124.19 °C ± 0.66, which was 0.54% lower compared to that of the neat LLDPE bottle (124.87 °C ± 0.54), as shown in Figure 9 and Table 1; a *T_c_* of 105.84 °C ± 0.93, which was 1.04% higher compared to that of the neat LLDPE bottle (104.75 °C ± 1.32), as shown in Figure 9 and Table 1; a crystallinity (%) of 37.05% ± 0.82, which was 0.11% higher compared to that of the neat LLDPE bottle (37.01% ± 1.25), as shown in Table 1; the temperature at which 5% weight loss occurred was 417.58 °C ± 1.31, which was 3.87% higher than that of the neat LLDPE (417.58 °C ± 1.31), as shown in Figure 10 and Table 2; and the estimated inorganic residual (wt.%) at around 600 (°C) was 8.67 ± 0.65, which was 1321% higher than that of the neat LLDPE (0.61 ± 0.11), as shown in Figure 10 and Table 2. Overall, the anti-microbial NPs did not significantly impact the thermal properties of the LLDPE extrusion blow-molded bottles.

The LLDPE extrusion blow-molded bottles thermally embossed with Cu-infused Mg(OH)_2_ (spray) showed the following **tensile properties**: the tensile stress at yield (MPa) was 8.37 ± 0.51, which was 3.79% higher compared to that of the neat LLDPE (8.06 ± 0.48); the tensile stress at break (MPa) was 20.81 ± 3.54, which was 9.82% higher compared to that of the neat LLDPE (18.95 ± 3.01); the tensile stress modulus (MPa) was 185.51 ± 8.11, which was 1.09% higher compared to that of the neat LLDPE (183.59 ± 7.05); and the elongation at break (%) was 704.63 ± 10.40, which was 0.33% lower compared to that of the neat LLDPE (706.99 ± 29.79). Overall, the anti-microbial NPs slightly improved the tensile properties, as shown in Figure 11. In some cases, especially when dealing with epoxy coatings, the incorporation of inorganic NPs can improve the mechanical properties of the polymeric matrices [49]. Impact strength and stiffness, in particular, were enhanced by the filling of potential pinholes and voids in the matrix.

The LLDPE extrusion blow-molded bottles thermally embossed with Mg(OH)_2_ (powder) showed the following **tensile properties**: the tensile stress at yield (MPa) was 8.77 ± 0.31, which was 8.80% higher compared to that of the neat LLDPE (8.06 ± 0.48); the tensile stress at break (MPa) was 20.40 ± 4.44, which was 7.65% higher compared to that of the neat LLDPE (18.95 ± 3.01); the tensile stress modulus (MPa) was 200.76 ± 8.31, which was 9.35% higher compared to that of the neat LLDPE (183.59 ± 7.05); and the elongation at break (%) was 692.03 ± 57.85, which was 2.12% lower compared to that of the neat LLDPE (706.99 ± 29.79). Overall, the anti-microbial NPs slightly improved the tensile properties, as presented in Figure 11.

The LLDPE extrusion blow-molded bottles thermally embossed with Mg(OH)_2_ (spray) showed the following **tensile properties**: the tensile stress at yield (MPa) was 8.04 ± 0.92, which was 0.26% lower compared to that of the neat LLDPE (8.06 ± 0.48); the tensile stress at break (MPa) was 18.30 ± 2.82, which was 3.43% lower compared to that of the neat LLDPE (18.95 ± 3.01); the tensile stress modulus (MPa) was 179.38 ± 18.91, which was 2.29% lower compared to that of the neat LLDPE (183.59 ± 7.05); and the elongation at break (%) was 705.47 ± 30.15, which was 0.21% lower compared to that of the neat LLDPE (706.99 ± 29.79). Overall, the anti-microbial NPs slightly improved the tensile properties, as presented in Figure 11.

The LLDPE extrusion blow-molded bottles thermally embossed with MgO (spray) showed the following **tensile properties**: the tensile stress at yield (MPa) was 8.49 ± 0.44, which was 5.32% higher compared to that of the neat LLDPE (8.06 ± 0.48); the tensile stress at break (MPa) was 20.36 ± 3.76, which was 7.44% higher compared to that of the neat LLDPE (18.95 ± 3.01); the tensile stress modulus (MPa) was 193.21 ± 15.46, which was 5.24% higher compared to that of the neat LLDPE (183.59 ± 7.05); and the elongation at break (%) was 692.45 ± 28.40, which was 2.01% lower compared to that of the neat LLDPE (706.99 ± 29.79). Overall, the anti-microbial NPs slightly improved the tensile properties, as presented in Figure 11.

The LLDPE extrusion blow-molded bottles thermally embossed with Cu(OH)_2_ (spray) showed the following **tensile properties**: the tensile stress at yield (MPa) was 8.07 ± 0.54, which was 0.11% higher compared to that of the neat LLDPE (8.06 ± 0.48); the tensile stress at break (MPa) was 19.62 ± 2.66, which was 3.54% higher compared to that of the neat LLDPE (18.95 ± 3.01); the tensile stress modulus (MPa) was 176.74 ± 9.96, which was 3.73% lower compared to that of the neat LLDPE (183.59 ± 7.05); and the elongation at break (%) was 695.96 ± 31.80, which was 1.56% lower compared to that of the neat LLDPE (706.99 ± 29.79). Overall, the anti-microbial NPs slightly improved the tensile properties, as presented in Figure 11.

The LLDPE extrusion blow-molded bottles thermally embossed with ZnO (spray) showed the following **tensile properties**: the tensile stress at yield (MPa) was 8.71 ± 0.66, which was 8.05% higher compared to that of the neat LLDPE (8.06 ± 0.48); the tensile stress at break (MPa) was 20.95 ± 4.01, which was 10.55% higher compared to that of the neat LLDPE (18.95 ± 3.01); the tensile stress modulus (MPa) was 198.10 ± 12.84, which was 7.90% higher compared to that of the neat LLDPE (183.59 ± 7.05); and the elongation at break (%) was 710.27 ± 7.11, which was 0.46% higher compared to that of the neat LLDPE (706.99 ± 29.79). Overall, the anti-microbial NPs slightly improved the tensile properties, as presented in Figure 11.

The effects of the anti-microbial agents, the variation in tensile bar weight, and the variation in the tensile bar thickness were statistically investigated to study their possible impacts on the tensile properties of the extrusion blow-molded bottles. The introduction of these anti-microbial agents at these loading levels (10,000 ppm and five sprays on each side of the mold cavity) can be achieved without any impact on the tensile properties, while providing a significant anti-microbial property to the bottles. The statistical analysis showed that after adjusting for the variation attributed to tensile bar thickness and bar weight, none of the six types of anti-microbial agents exhibited significantly different results to those of the control, as measured by tensile stress at yield, tensile stress at break, modulus, and elongation at break. A detailed statistical analysis is provided as part of the SI document (please see Section S4).

The LLDPE extrusion blow-molded bottles thermally embossed with Cu-infused Mg(OH)_2_ (spray) showed the following **barrier properties**: the WVTR (g/(m^2^·day)) and OTR (cm^3^/(m^2^·day)) were 13.34 ± 1.56 and 1037.56 ± 76.46, respectively, which were 26.54% and 9.63% lower, respectively, compared to that of the neat LLDPE (18.16 ± 1.93 and 1148.11 ± 18.84). Overall, the anti-microbial NPs improved the barrier properties, as presented in Figure 12.

The LLDPE extrusion blow-molded bottles thermally embossed with Mg(OH)_2_ (powder) showed the following **barrier properties**: the WVTR (g/(m^2^·day)) and OTR (cm^3^/(m^2^·day)) were 16.32 ± 0.63 and 1000.95 ± 62.25, respectively, which were 10.13% and 12.82% lower, respectively, compared to that of the neat LLDPE (18.16 ± 1.93 and 1148.11 ± 18.84). Overall, the anti-microbial NPs improved the barrier properties, as presented in Figure 12.

The LLDPE extrusion blow-molded bottles thermally embossed with Cu-infused Mg(OH)_2_ (spray) showed the following **barrier properties**: the WVTR (g/(m^2^·day)) and OTR (cm^3^/(m^2^·day)) were 13.67 ± 0.62 and 463.50 ± 41.29, respectively, which were 24.72% and 59.63% lower, respectively, compared to that of the neat LLDPE (18.16 ± 1.93 and 1148.11 ± 18.84). Overall, the anti-microbial NPs improved the barrier properties, as presented in Figure 12.

The LLDPE extrusion blow-molded bottles thermally embossed with MgO (spray) showed the following **barrier properties**: the WVTR (g/(m^2^·day)) and OTR (cm^3^/(m^2^·day)) were 14.43 ± 0.59 and 1018.70 ± 67.63, respectively, which were 20.54% and 11.27% lower, respectively, compared to that of the neat LLDPE (18.16 ± 1.93 and 1148.11 ± 18.84). Overall, the anti-microbial NPs improved the barrier properties, as presented in Figure 12.

The LLDPE extrusion blow-molded bottles thermally embossed with Cu(OH)_2_ (spray) showed the following **barrier properties**: the WVTR (g/(m^2^·day)) and OTR (cm^3^/(m^2^·day)) were 14.52 ± 0.37 and 537.29 ± 70.92, respectively, which were 20.04% and 53.20% lower, respectively, compared to that of the neat LLDPE (18.16 ± 1.93 and 1148.11 ± 18.84). Overall, the anti-microbial NPs improved the barrier properties, as presented in Figure 12.

The LLDPE extrusion blow-molded bottles thermally embossed with ZnO (spray) showed the following **barrier properties**: the WVTR (g/(m^2^·day)) and OTR (cm^3^/(m^2^·day)) were 16.81 ± 0.50 and 937.25 ± 64.30, respectively, which were 7.43% and 18.37% lower, respectively, compared to that of the neat LLDPE (18.16 ± 1.93 and 1148.11 ± 18.84). Overall, the anti-microbial NPs improved the barrier properties, as presented in Figure 12.

The improved barrier properties were likely due to the fixation of the inorganic crystals over the outer surface of the extrusion blow-molded bottles. Upon the incorporation of these particles, the porosities of the bottles were considerably narrowed. In all cases, the coating improved the gas barrier properties of the bottle. While the improvement of WVTR is only marginal in most cases, the improvement of OTR for sample (Figure 12D,F) is quite noticeable, with ~50% improvement. It is known that inorganic-based coatings can improve gas barrier performance [50]. In this case, the coating was not specifically tuned to improve gas barrier performance, hence the marginal improvement.

The **anti-microbial performance** of extrusion blow-molded bottles was tested against *E. coli* K-12 MG1655 (8.16 ± 0.10 log), as presented in Figure 13. The metallic copper disks (positive control) showed a 6.52 ± 0.07 and 8.16 ± 0.07 log reduction at 4 h and 24 h, respectively. The neat LLDPE disks (negative control) showed a 0.25 ± 0.03 and 0.66 ± 0.12 log reduction at 4 h and 24 h, respectively. The negative control’s performance shows a bacterial reduction too low to be considered antibacterial and in combination with the positive control’s result, this constitutes proof of the validity of the anti-bacterial test. The extrusion blow-molded bottles thermally embossed with Cu-infused Mg(OH)_2_ particles (spray) showed a 4.11 ± 0.20 and 8.09 ± 0.07 log reduction at 4 h and 24 h, respectively, which presented a 99.999996 and 99.999996% reduction from the negative control, respectively. The extrusion blow-molded bottles thermally embossed with Mg(OH)_2_ particles (powder) showed a 3.02 ± 0.07 and 5.43 ± 0.03 log reduction at 4 h and 24 h, respectively, which exhibited a 99.8333336 and 99.999996% reduction from the negative control, respectively. The extrusion blow-molded bottles thermally embossed with Mg(OH)_2_ particles (spray) showed a 4.04 ± 0.30 and 8.00 ± 0.05 log reduction at 4 h and 24 h, respectively, which presented a 99.988886 and 99.999996% reduction from the negative control, respectively. The extrusion blow-molded bottles thermally embossed with MgO particles (spray) showed a 3.95 ± 0.02 and 8.20 ± 0.07 log reduction at 4 h and 24 h, respectively, which was a 99.988886 and 99.999996% reduction from the negative control, respectively. The extrusion blow-molded bottles thermally embossed with Cu(OH)_2_ particles (spray) showed a 3.88 ± 0.09 and 7.32 ± 0.04 log reduction at 4 h and 24 h, respectively, which showed a 99.988886 and 99.999996% reduction from the negative control, respectively. The extrusion blow-molded bottles thermally embossed with ZnO particles (spray) showed a 1.40 ± 0.08 and 3.32 ± 0.10 log reduction at 4 h and 24 h, respectively, which was a 92.9222226 and 99.7888886% reduction from the negative control, respectively.

In all cases, the bacterial reduction after 24 h is highly increased compared to that at 4 h, showing that these coatings require several hours to a day to fully eradicate the initial incubated bacterial colonies. The ZnO reflects the lowest performance, with only a log 3 reduction after 24 h. ZnO NPs are known to be effective anti-bacterial agents, but superior additives, such as Ag NPs, are available [51]. For instance, Mg(OH)_2_ powder provided better performance, reaching a log 5.5 bacterial reduction after 24 h. The performance was further enhanced when applying Cu- or Mg-based additives via the spray coating (Figure 13C,E–G); all have a bacterial reduction > to log 7 after 24 h. This level of sterility is far superior to that provided by disinfection and can only be achieved through sterilization techniques such as gamma radiation [52].

## 5. Conclusions

In this article, a novel method toward the development of anti-microbial extrusion blow-molded LLDPE bottles was reported, in which various types of anti-microbial agents (Cu-infused Mg(OH)_2_, Mg(OH)_2_, Cu(OH)_2_, MgO, CuCl_2_, and ZnO) were introduced onto the surface of the bottles. The produced samples were characterized via SEM and EDX and were evaluated for their thermal, mechanical, and anti-microbial properties via DSC, TGA, tensile, barrier, and anti-microbial testing. The results demonstrate significant improvement in anti-microbial activities, as well as barrier properties, while maintaining the thermal stability and mechanical performance of the neat polymer. This approach might be useful in industrial scale applications to help create environmentally friendly, cost-effective, time-efficient, and easily implemented anti-microbial systems. This study provides a promising alternative to the conventional melt-compounding process, in which the anti-microbial agents are mixed with plastic materials during the manufacturing process, requiring a higher load percentage of additives.

## Figures and Tables

**Table 1:** Thermal properties of various LLDPE samples obtained from DSC measurements.

Samples	Δ*H*_m_ [J/g]	*T*_m_ [°C]	*T*_c_ [°C]	*Crystallinity* [%]
Neat LLDPE	102.90 ± 1.43	124.87 ± 0.54	104.75 ± 1.32	37.01 ± 1.25
LLDPE Cu-infused Mg(OH)_2_ (Spray)	96.81 ± 0.99	124.23 ± 0.10	105.87 ± 0.87	34.82 ± 1.77
LLDPE Mg(OH)_2_ (Powder)	98.25 ± 1.87	123.97 ± 1.54	106.01 ± 1.44	35.34 ± 0.87
LLDPE Mg(OH)_2_ (Spray)	106.80 ± 1.78	123.91 ± 0.76	106.12 ± 0.45	38.41 ± 0.93
LLDPE MgO (Spray)	99.09 ± 0.81	123.78 ± 0.37	105.75 ± 0.44	35.64 ± 1.34
LLDPE Cu(OH)_2_ (Spray)	113.40 ± 1.12	124.58 ± 0.88	105.48 ± 1.61	40.79 ± 1.21
LLDPE ZnO (Spray)	103.00 ± 1.09	124.19 ± 0.66	105.84 ± 0.93	37.05 ± 0.82

**Table 2:** The temperature at which various LLDPE extrusion blow-molded bottles underwent 5% weight loss, and the estimated residual at 600 (°C), as determined via TGA measurements.

Sample	The Temperature at Which 5% Weight Loss Occurred (°C)	Estimated Residual (wt.%) at 600 (°C)
Neat LLDPE	402.03 ± 1.62	0.61 ± 0.11
LLDPE Cu-infused Mg(OH)_2_ (Spray)	413.59 ± 0.86	0.00 ± 0.43
LLDPE Mg(OH)_2_ (Powder)	441.28 ± 1.98	0.12 ± 0.82
LLDPE Mg(OH)_2_ (Spray)	426.01 ± 2.32	4.91 ± 0.08
LLDPE MgO (Spray)	422.12 ± 1.11	6.56 ± 0.45
LLDPE Cu(OH)_2_ (Spray)	425.96 ± 0.95	0.03 ± 1.22
LLDPE ZnO (Spray)	417.58 ± 1.31	8.67 ± 0.65

## Data Availability

All data are available in the SI document.

## Supplementary Materials

The following supporting information can be downloaded at: https://www.mdpi.com/article/10.3390/polym16131914/s1, Figure S1: Illustration of the extrusion blow molding process; Figure S2: Extrusion blow molding machine and model specifications; Figure S3: Bottle mold design; Figure S4: Embossing powder tool used for Mg(OH)_2_ NPs (dry powder) deposition into the mold cavity; Figure S5: The bottle mold after the application/deposition of the Mg(OH)_2_ dry powder; Figure S6: EDX Mapping for LLDPE bottle thermally embossed with Cu-infused Mg(OH)_2_ (Spray): SEM image of sample (A), O element mapping (B), Mg element mapping (C), Cl element mapping (D), and Cu element mapping (E); Figure S7: EDX Mapping for LLDPE bottle thermally embossed with Mg(OH)_2_ (powder): SEM image of sample (A), C element mapping (B), O element mapping (C), Mg element mapping (D), and Al element mapping (E); Figure S8: EDX Mapping for LLDPE bottle thermally embossed with Mg(OH)_2_ (spray): SEM image of sample (A), C element mapping (B), O element mapping (C), and Mg element mapping (D); Figure S9: EDX Mapping for LLDPE bottle thermally embossed with MgO (spray): SEM image of sample (A), C element mapping (B), O element mapping (C), Mg element mapping (D), and Ca element mapping; Figure S10: EDX Mapping for LLDPE bottle thermally embossed with Cu(OH)2 (spray): SEM image of sample (A), C element mapping (B), O element mapping (C), Al element mapping (D), Cl element mapping (E), and Cu element mapping (F); Figure S11: EDX Mapping for LLDPE bottle thermally embossed with ZnO (spray): SEM image of sample (A), C element mapping (B), O element mapping (C), and Zn element mapping (D); Figure S12: Comparison with control decision chart; Figure S13: Comparison with control decision chart; Figure S14: Comparison with control decision chart; Figure S15: Comparison with control decision chart; Table S1: Table S1: Results from stepwise regression on noise factors for each of the outcomes, tensile stress at yield, tensile stress at break, tensile modulus, and elongation at break. *p*-values less than 0.05 are in italics; Table S2: Parameter estimates; Table S3: Comparisons with control summary; Table S4: Parameter estimates; Table S5: Comparison with control summary; Table S6: Parameter estimates; Table S7: Comparisons with control summary; Table S8: Parameter estimates; Table S9: Comparisons with control summary.
