# Supplementary material for: Anti-Microbial, Thermal, Mechanical, and Gas Barrier Properties of Linear Low-Density Polyethylene Extrusion Blow-Molded Bottles
Source: Polymers (Basel). 2024 Jul 4;16(13):1914. doi: 10.3390/polym16131914 (PMC11244499; doi:10.3390/polym16131914)
Supplement: Supplementary file 1 [file polymers-16-01914-s001.zip › polymers-3050979-supplementary.pdf]

polymers

Supplementary Information

Anti-Microbial, Thermal, Mechanical, and Gas Barrier Properties of Linear Low-Density Polyethylene Extrusion Blow-Molded Bottles

Saleh Alkarri [1,*], Muhammed Naveed [1], Fatimah Alali [2], Jérôme Vachon [3], Aaron Walworth [1] and Abigail Vanderberg [4]

1 School of Packaging, Michigan State University, 448 Wilson Road, East Lansing, MI 48824-1223, USA
2 Almoosa College of Health Sciences, Ain Najm Rd, Al Mubarraz 36422, Saudi Arabia
3 SABIC, Urmonderbaan 22, 6167 RD Geleen, P.O. Box 319, 6160 AH Geleen, The Netherlands
4 Center for Advanced Microscopy, Michigan State University, 578 Wilson Road, CIPS Bldg, Rm B-6B, East Lansing, MI48824-1223, USA
* Correspondence: alkarris@msu.edu

Contents of the SI

- S1. Extrusion Blow Molder (Figures S1 – S3)
- S2. Dry Powdered-Nanoparticle Applicator (Figures S4 and S5)
- S3. EDX Maping (Figures S6 – S11)
- S4. Statistical Analysis of The Tensile Properties of The LLDPE Extrusion Blow Molded Bottles (Tables S1 – S9 and Figures S12 – S15)

S1. Extrusion Blow Molder:

The following highlights the process (seen in **Figure S1**) and specifications of the extrusion blow molding instrument (seen in **Figure S2**) used to produce the five LLDPE bottles studied in this article. A Bekum H111S extrusion blow molder (Serial No. 974948-5-056) produced in 2001 has the following specifications: dual electrical feed of 480 volt and 208 volt, circuit breaker size of 100 and 100 AMPS, an interrupt capacity of 65k and 18k AMPS, calculated full-load amperage (FLA) of 56 and 41 AMPS, largest load FLA of 22 and 8 AMPS, control circuit of 24 VDC and an electrical/electricity diagram number of 975361. The instrument's bottle mold design is presented in (**Figure S3**).

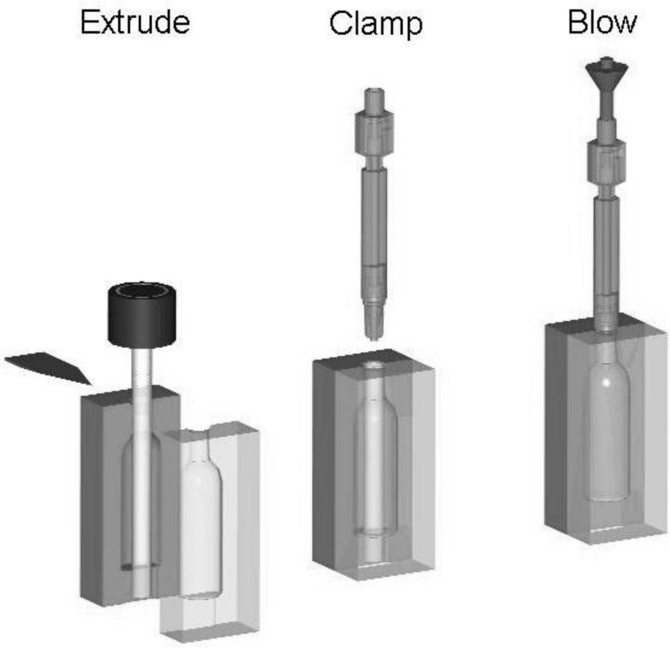

Figure S1. Illustration of the extrusion blow molding process[1].

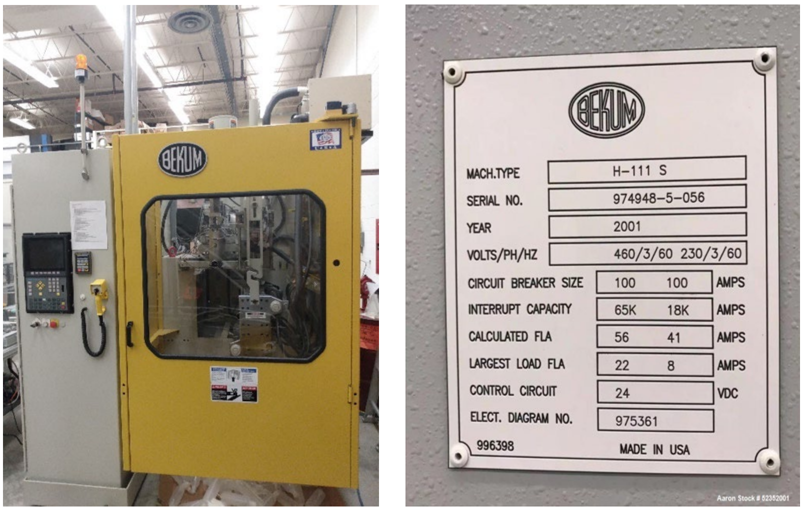

Figure S2. Extrusion blow molding machine and model specifications.

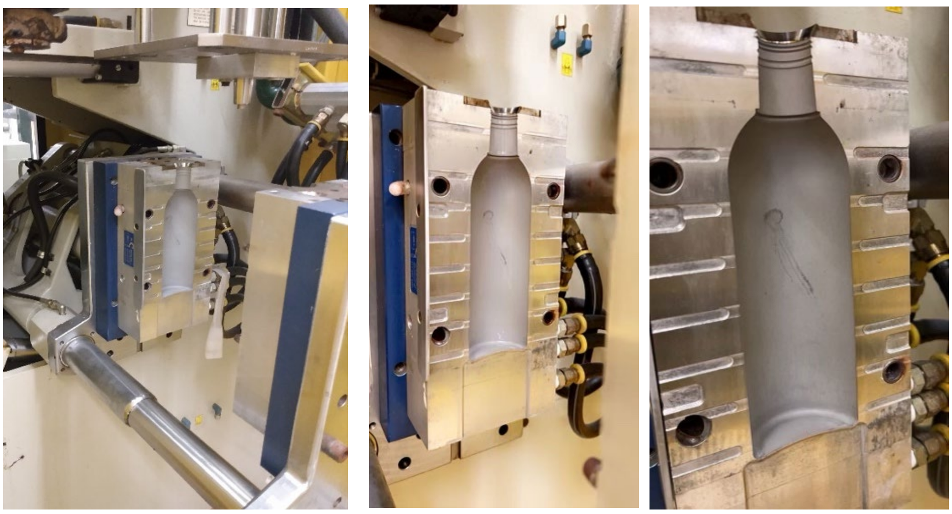

Figure S3. Bottle mold design.

S2. Dry Powder-Nanoparticle Applicator

A cosmetic embossing powder tool (Brand: BAOFALI) was first used as an applicator of the dry powdered nanoparticles ($Mg(OH)_2$) onto the bottle mold cavity before the start of each production cycle (**Figure S4**). The mold after the deposition of the dry powder nanoparticles is shown for reference in (**Figure S5**).

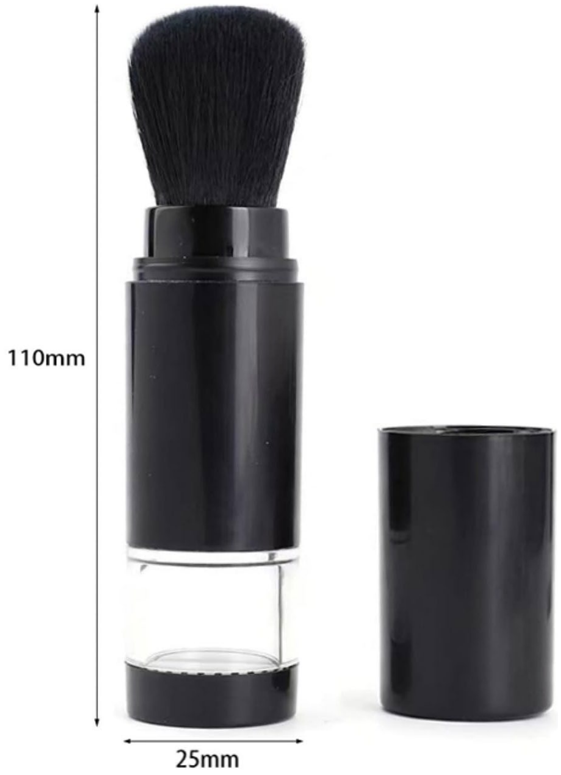

Figure S4. Embossing powder tool used for Mg(OH)2 NPs (dry powder) deposition into the mold cavity.

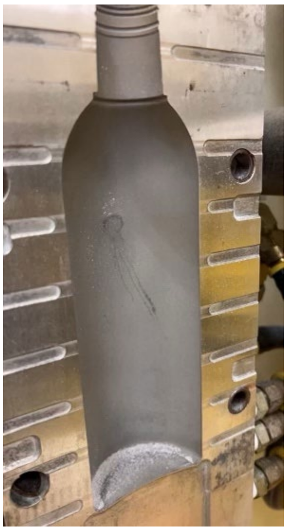

Figure S5. The bottle mold after the application/deposition of the $Mg(OH)_2$ dry powder.

S3. EDX Mapping:

During SEM analysis, EDX mapping data was collected to show the distribution of nanoparticles on the surface of thermally embossed blow molded bottles. Map analysis areas measured approximately 13 µm by 10 µm. Samples were mapped using an using an Oxford Instruments AZtec system (Oxford Instruments, High Wycomb, Bucks, England).

S3.1 Cu-infused $Mg(OH)_2$ (Spray):

Mapping images of LLDPE extrusion blow molded bottles thermally embossed with Cu-infused $Mg(OH)_2$ (spray) are shown in (**Figure S6**).

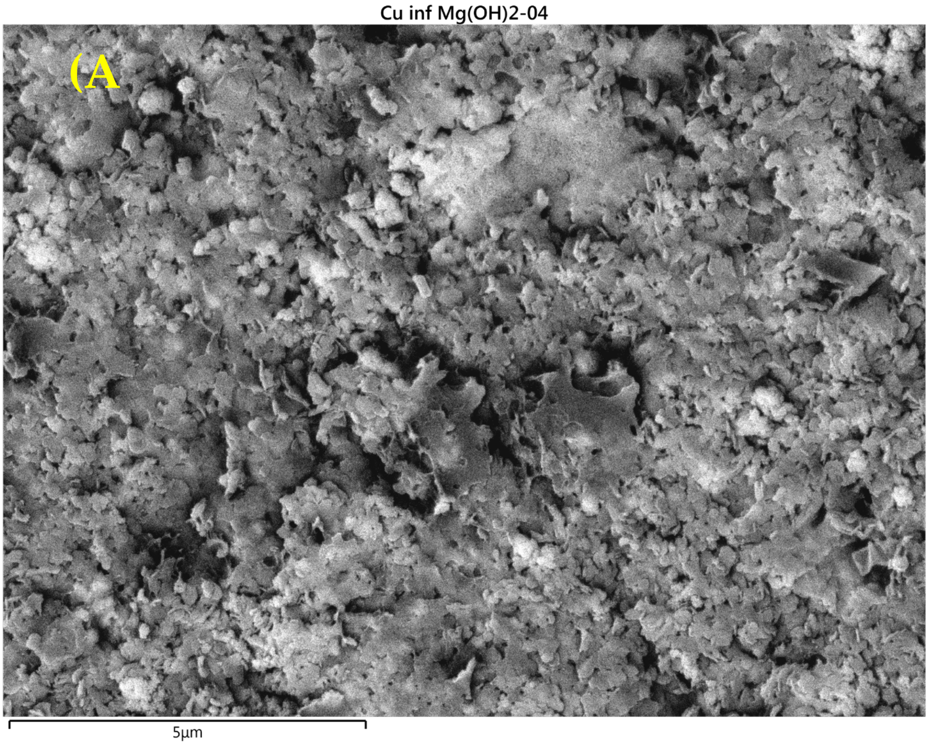

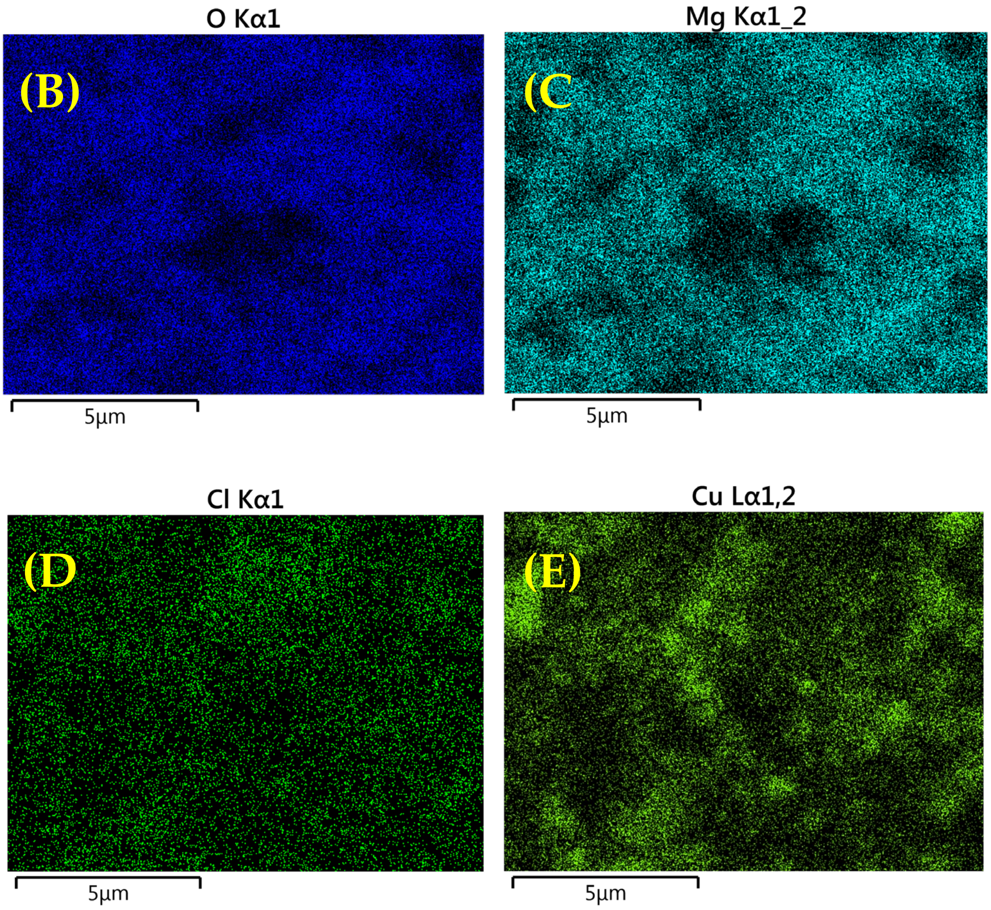

Figure S6. EDX Mapping for LLDPE bottle thermally embossed with Cu-infused $Mg(OH)_2$ (Spray): SEM image of sample (A), O element mapping (B), Mg element mapping (C), Cl element mapping (D), and Cu element mapping (E).

S3.2 $Mg(OH)_2$ Powder:

Mapping images of LLDPE extrusion blow molded bottles thermally embossed with $Mg(OH)_2$ (powder) are shown in (**Figure S7**).

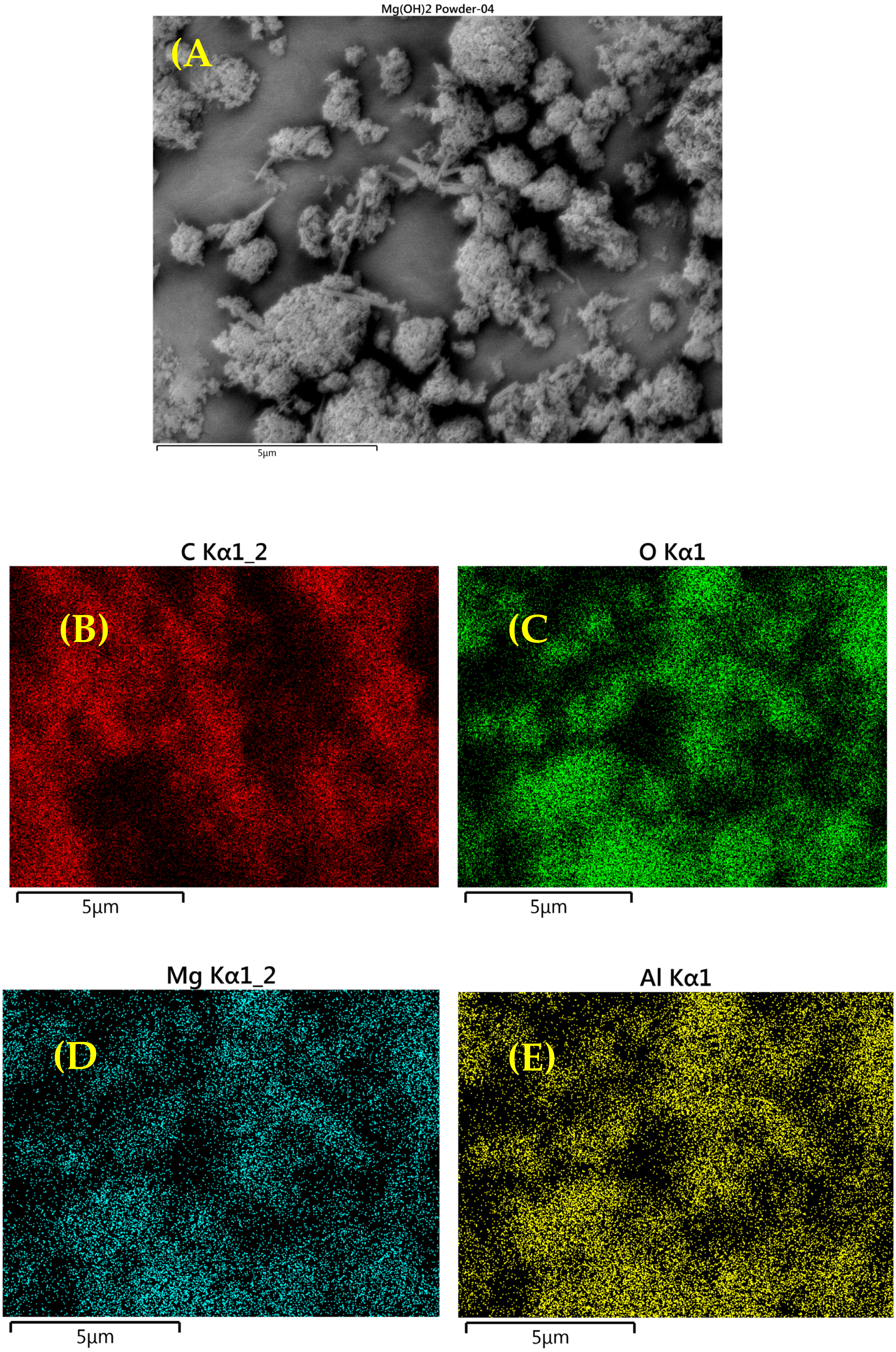

Figure S7. EDX Mapping for LLDPE bottle thermally embossed with $Mg(OH)_2$ (powder): SEM image of sample (A), C element mapping (B), O element mapping (C), Mg element mapping (D), and Al element mapping (E).

S3.3 $Mg(OH)_2$ (Spray):

Mapping images of LLDPE extrusion blow molded bottles thermally embossed with $Mg(OH)_2$ (spray) are shown in (**Figure S8**).

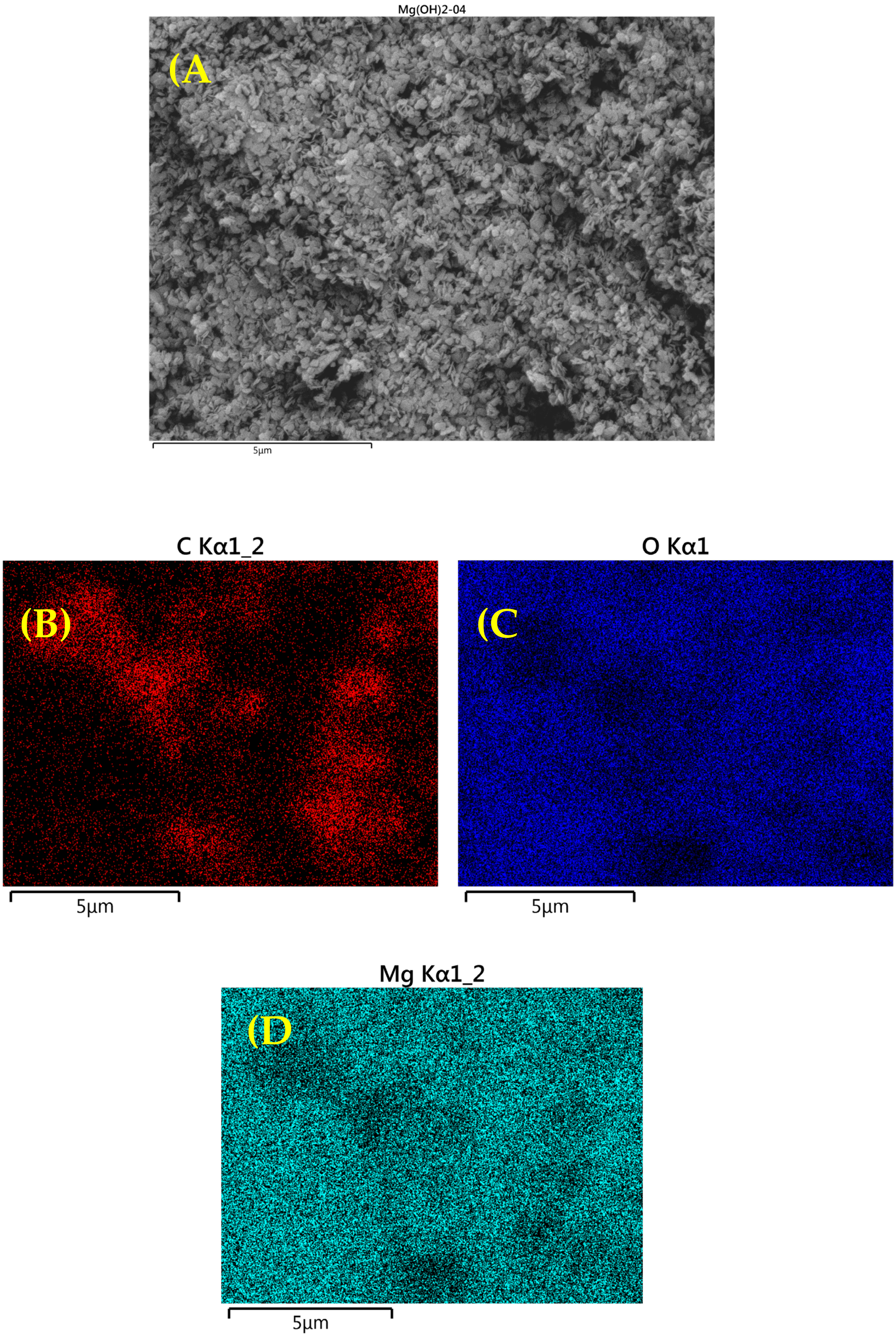

Figure S8. EDX Mapping for LLDPE bottle thermally embossed with $Mg(OH)_2$ (spray): SEM image of sample (A), C element mapping (B), O element mapping (C), and Mg element mapping (D).

S3.4 MgO (spray):

Mapping images of LLDPE extrusion blow molded bottles thermally embossed with MgO (spray) are shown in (**Figure S9**).

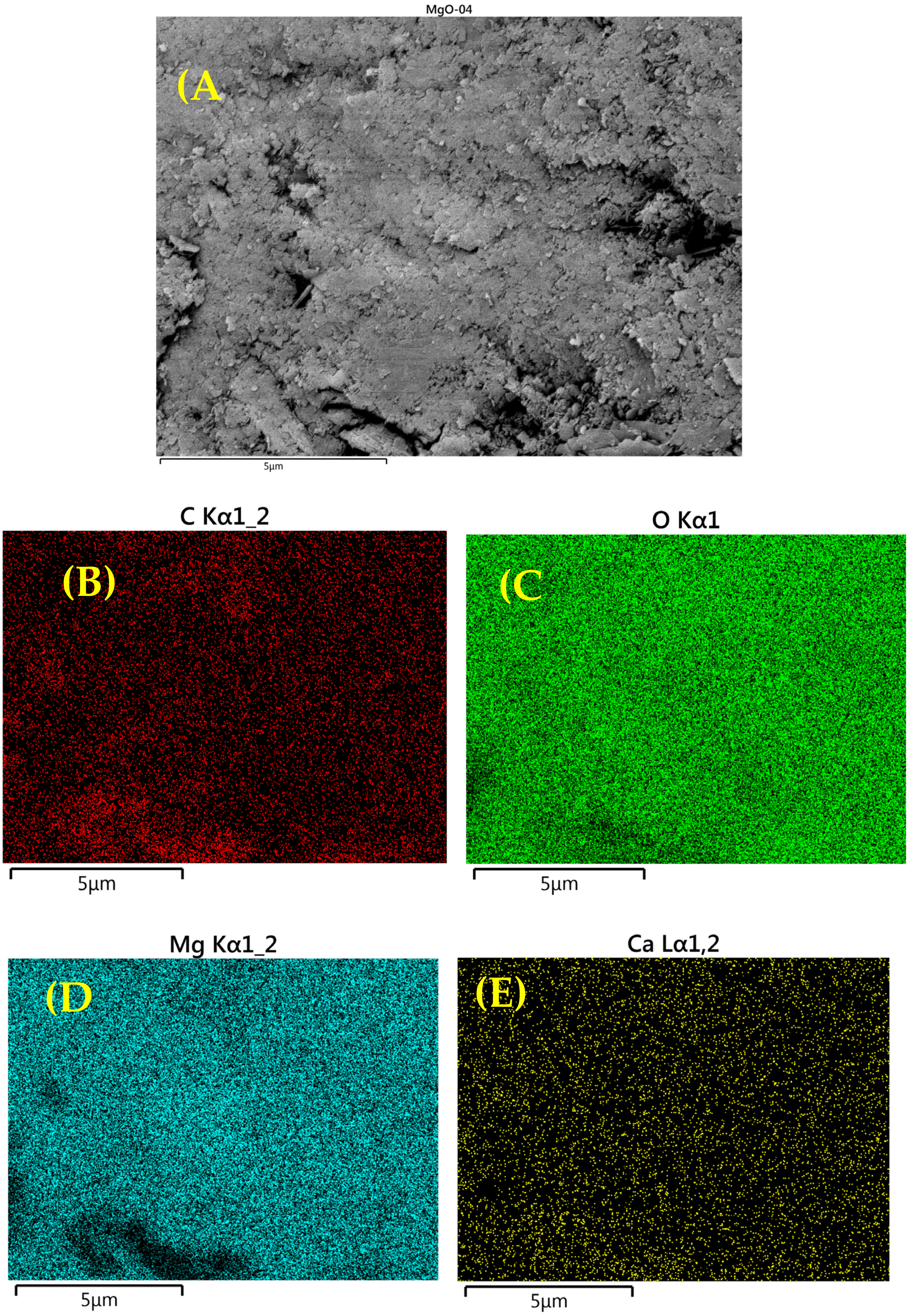

Figure S9. EDX Mapping for LLDPE bottle thermally embossed with MgO (spray): SEM image of sample (A), C element mapping (B), O element mapping (C), Mg element mapping (D), and Ca element mapping.

S3.5 $Cu(OH)_2$ (spray):

Mapping images of LLDPE extrusion blow molded bottles thermally embossed with $Cu(OH)_2$ (spray) are shown in (**Figure S10**).

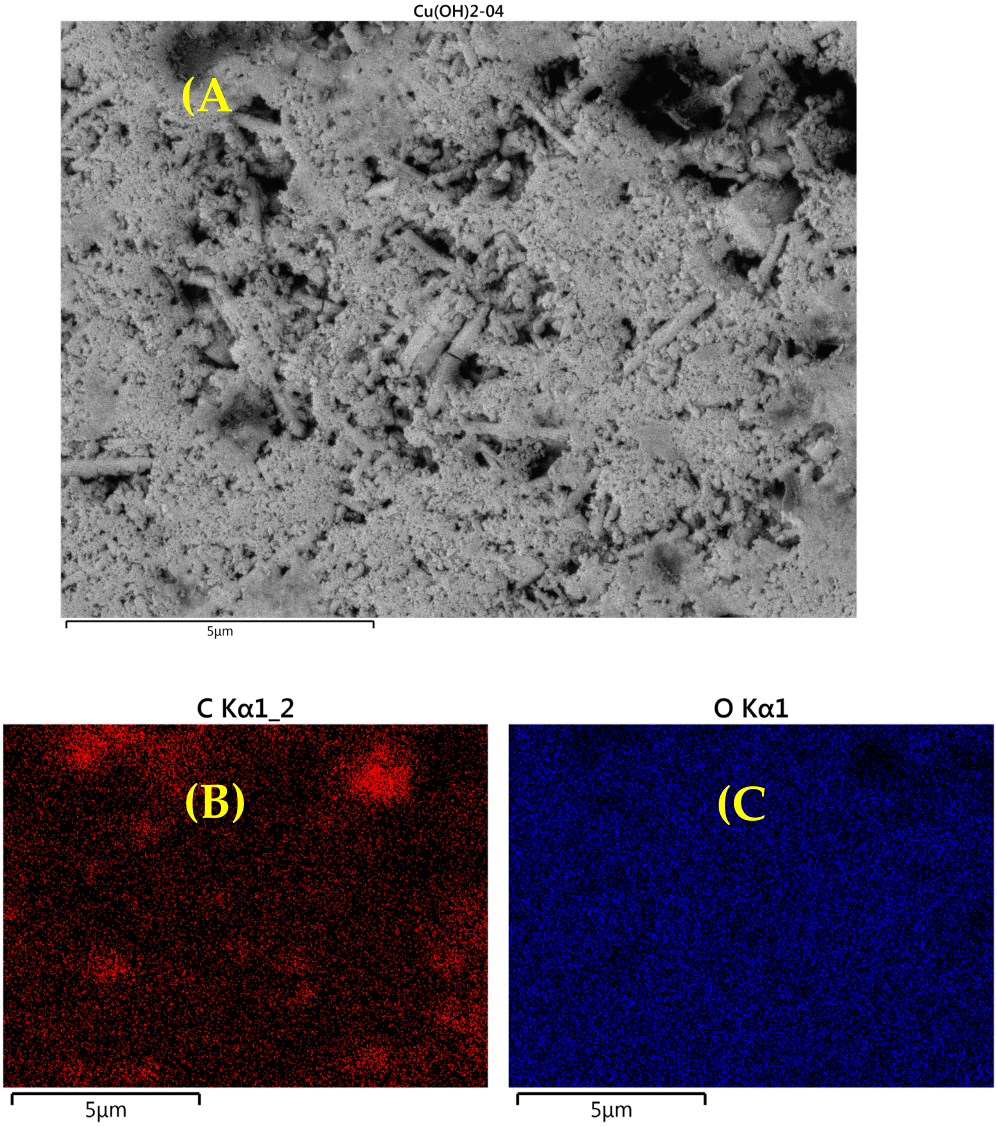

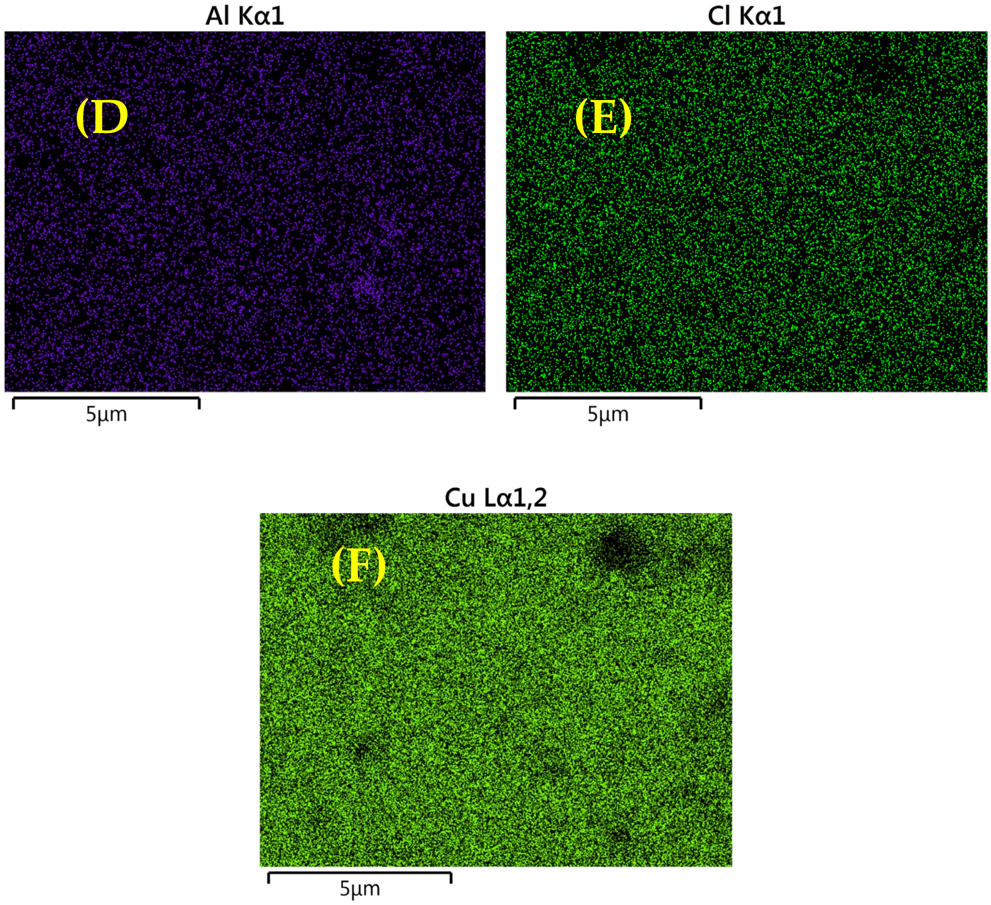

Figure S10. EDX Mapping for LLDPE bottle thermally embossed with $Cu(OH)_2$ (spray): SEM image of sample (A), C element mapping (B), O element mapping (C), Al element mapping (D), Cl element mapping (E), and Cu element mapping (F).

S3.6 ZnO (spray):

Mapping images of LLDPE extrusion blow molded bottles thermally embossed with ZnO (spray) are shown in (**Figure S11**).

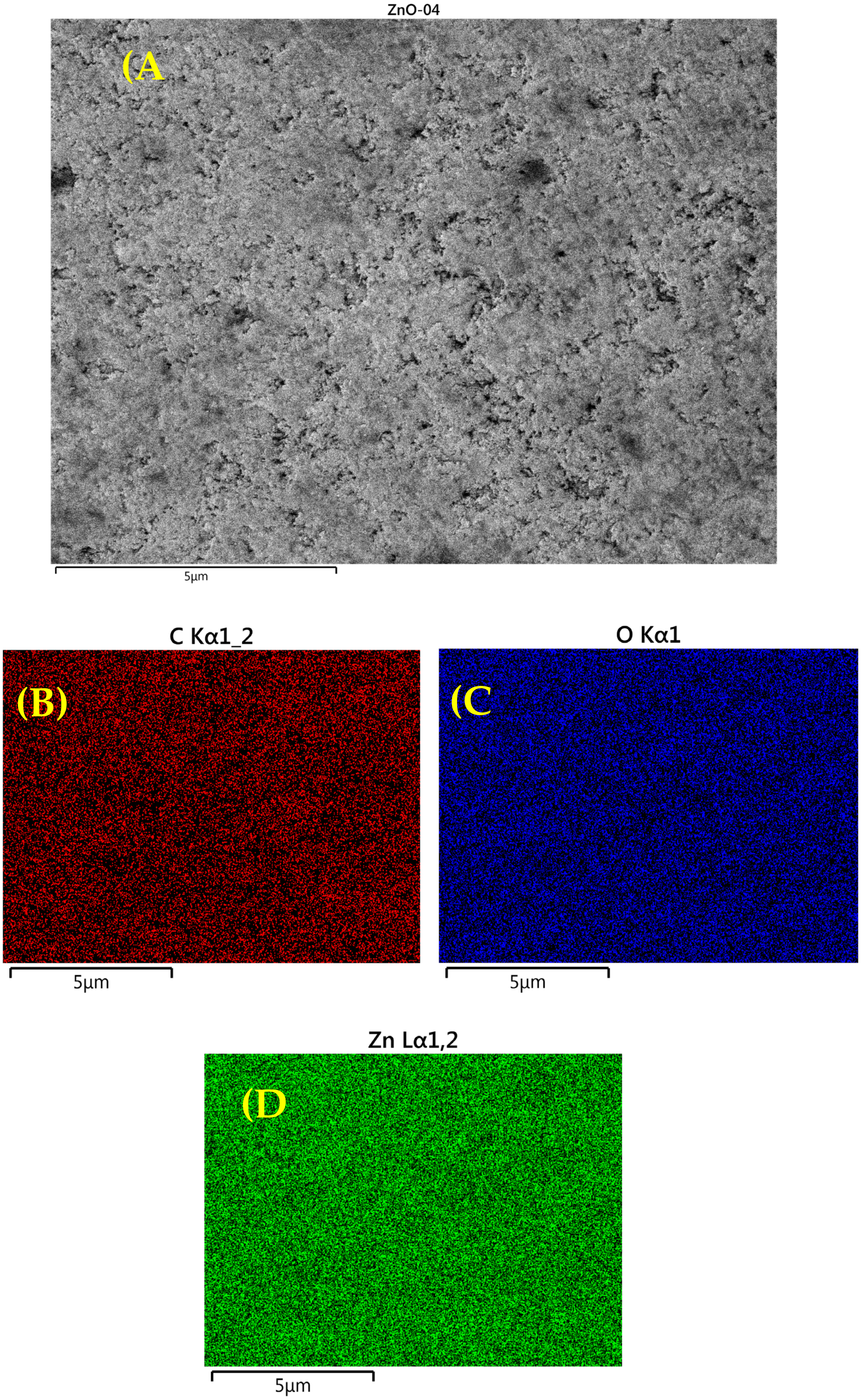

Figure S11. EDX Mapping for LLDPE bottle thermally embossed with ZnO (spray): SEM image of sample (A), C element mapping (B), O element mapping (C), and Zn element mapping (D).

S4. Statistical Analysis of The Tensile Properties of The LLDPE Extrusion Blow Molded Bottles

S4.1 Objective

Investigate the effect of anti-microbial agents (Cu-infused $Mg(OH)_2$ spray, $Mg(OH)_2$ powder**,** $Mg(OH)_2$ spray, MgO spray, $Cu(OH)_2$ spray, and ZnO spray) on the tensile properties of extrusion blow molded bottles while accounting for variablity in tensile bar weight and tensile bar thickness that are caused by the experiment's methods (conventional extrusion blow molding).

S4.2 Introduction

The goal of this set of experiments was to study the effects of applying six types of anti-microbial agents on the tensile properties (four outcomes) of extrusion blow molded bottles. The extrusion blow molded LLDPE bottles were thermally embossed with various types of anti-microbial agents: Cu-infused $Mg(OH)_2$ (spray), $Mg(OH)_2$ (Powder), $Mg(OH)_2$ (spray), MgO (spray), $Cu(OH)_2$ (spray), and ZnO (spray). The thermally embossed bottles with various anti-microbial agents had been compared to a control preparation of neat LLDPE bottles. The studied four outcomes were (i) tensile stress at yield (MPa), (ii) tensile stress at break (MPa), (iii) tensile modulus (MPa), and (iv) elongation at break (%).

S4.3 Data Analysis Method

Five replicates of each agent were tested for a total of 35 experiments. The experiments were not conducted in a randomized order; therefore, there is an opportunity to improve the methodology and reduce the number of experiments required if this experiment is to be repeated in the future. An optimal design of experiment (DoE) was created in JMP software (JMP Pro 16.1.0 (539038), SAS Institute Inc, Cary, North Carolina, USA) with seven categorical factors, two uncontrolled factors, two replicate runs, and a response surface model for analysis. This optimal design would reduce the number of experiments from 35 to 28.

S4.4 Noise Factors

The two potential sources of noise that may interfere with this analysis of anti-microbial agents have been identified as (i) variability in the thickness of the tensile bar and (ii) variations in the tensile bar weight.

S4.5 Statistical Design

The potential noise factors have been evaluated individually, followed by analysis of variance (ANOVA), followed by multiple comparisons tests. Each of the four effects are evaluated with the ANOVA results form, a Least Squares Fit, and a multiple comparison using Dunnett's test through JMP software to compare each anti-microbial agent to the control.

S4.6 Statistical Analysis

The weight of each tensile bar was measured in (mg). The average bar weight across all samples was 0.988 mg with a standard deviation of 0.354 mg. The tensile bar thickness was measured at three points along the tensile bar's gage length. The two potential sources of noise are variations in the bar weight and variability in thickness of the tensile bar. The tensile bar weight (mg) of various LLDPE blow molded bottles thermally embossed with variety of anti-microbial agents were as follows: LLDPE control (1.01±0.41), LLDPE Cu-infused $Mg(OH)_2$ (spray) (0.98±0.34), LLDPE $Mg(OH)_2$ (powder) (0.99±0.38), LLDPE $Mg(OH)_2$ (spray) (1.13±0.47), LLDPE MgO (spray) (0.93±0.31), LLDPE $Cu(OH)_2$ (spray) (0.90±0.37), and LLDPE ZnO (spray) (0.98±0.39).

The average tensile bar thickness (μm) of each LLDPE blow bottle was measured at three points in each sample (M1, M2, and M3) of the gage length. The average tensile bar thickness across the control and thermally embossed bottles were as follows: LLDPE control (499.53±186.02), LLDPE Cu-infused $Mg(OH)_2$ (spray) (470.93±175.22), LLDPE $Mg(OH)_2$ (powder) (463.00±185.35), LLDPE $Mg(OH)_2$ (spray) (576.47±233.64), LLDPE MgO (spray) (446.93±169.26), LLDPE $Cu(OH)_2$ (spray) (440.87±174.52), and LLDPE ZnO (spray) (459.80±204.87).

In order to capture the variability in the thickness across the gage length of the bar, the coefficient in variation (CV) of these three thickness measurements was calculated for each experimental unit. The CV measures the variability of the measurements about the mean. The higher the CV, the more variable the measurements. The formula for CV was as follows:

$$CV = \frac{\sigma}{\mu}$$

Where σ was the standard deviation of the measurements and μ was the mean of the measurements. The average CV for the 35 samples is 5.7 % with a range of 21.1 %.

S4.7 Evaluation of Noise Factors

The noise factor presents a challenge because they were not orthogonal. There was a 35 % correlation between the tensile bar weight and CV. To handle this situation, a sequential ANOVA technique and the Incremental Sums of Squares were used. The plan was to construct the sequence of sub-models using stepwise forward selection followed by stepwise backwards selection to minimize the Bayesian Information Criterion (BIC). The BIC is a model comparison metric that assesses the model strength and accounts for model complexity. Stepwise selection indicates that the tensile bar weight is playing a role in the tensile stress at break ($p < 0.0001$) and the tensile modulus ($p = 0.0149$) as shown in (**Table S1**). In addition, CV was not needed explain any outcome.

Table S1. Results from stepwise regression on noise factors for each of the outcomes, tensile stress at yield, tensile stress at break, tensile modulus, and elongation at break. P-values less than 0.05 are in italics.

	T stress at yield	T stress at break	T modulus	Elongation at Break
Bar Weight	0.3315	*<.0001*	*0.0149*	0.1957
CV	0.5919	0.1718	0.4056	0.1937

Considering the results for the stepwise selection for each of the four outcomes, all analyses will adjust for the tensile bar weight before comparing the anti-microbial agents, and the CV of the samples will be disregarded.

S6.8 Response to Tensile Stress at Yield (MPa)

The tensile stress at yield was evaluated using the following model:

$$y = \mu + \tau_r + \beta + e$$

Where y was the tensile yield strength, μ was the overall mean of the tensile strength, τ_r was the treatment effect of the anti-microbial agent, β was the effect of bar weight, and e was the standard deviation.

The ANOVA for tensile stress at yield indicated that after adjusting for differences in tensile bar weight, the tensile stress at yield for each to the anti-microbial agent was not significantly different from the control. The p-values were as follows: LLDPE Cu-infused $Mg(OH)_2$ (spray) (p=0.9601), LLDPE $Mg(OH)_2$ (powder) (p=0.1017), LLDPE $Mg(OH)_2$ (spray) (p=0.1485), LLDPE MgO (spray) (p=0.5495), LLDPE $Cu(OH)_2$ (spray) (p=0.3049), and LLDPE ZnO (spray) (p=0.1580) as shown in (**Table S2**).

Table S2. Parameter estimates.

Term	Estimate	Std Error	t Ratio	Prob>|t|
Intercept	8.0143506	0.304265	26.34	<.0001*
Anti-Microbial Agent[LLDPE Cu-infused $Mg(OH)_2$ (spray)]	0.012347	0.24425	0.05	0.9601
Anti-Microbial Agent[LLDPE $Cu(OH)_2$ (spray)]	-0.256925	0.245644	-1.05	0.3049
Anti-Microbial Agent[LLDPE $Mg(OH)_2$ (Powder)]	0.4138634	0.24423	1.69	0.1017
Anti-Microbial Agent[LLDPE $Mg(OH)_2$ (spray)]	-0.368501	0.247742	-1.49	0.1485
Anti-Microbial Agent[LLDPE MgO (spray)]	0.1483636	0.244785	0.61	0.5495
Anti-Microbial Agent[LLDPE ZnO (spray)]	0.3546868	0.244234	1.45	0.1580
Bar weight (mg)	0.3470449	0.290841	1.19	0.2432

To further evaluate the possible effects of anti-microbial agents on the tensile properties of extrusion blow molded bottles, the Dunnet's method was used. This method was developed to compare a large number of means to a control. The earlier p-values (presented in **Table S2**) were for the evaluation of the overall treatment effect, not the large number of comparisons needed for means comparison. There are several comparison methods, but Dunnett's method was specifically useful for comparison against a control. The control for this particular study was the neat LLDPE bottles.

The results again showed that none of the anti-microbial agents was significantly different to the control. All values fall within upper and lower decision limits as calculated using Dunnett's test. The upper limit was calculated to be between (9.075 to 9.080) and the lower limit was between (7.027 to 7.032) as shown in (**Figure S12**). All estimates fall within this range (7.027 to 9.080). The estimates for each anti-microbial agent were as follows: LLDPE Cu-infused $Mg(OH)_2$ (spray) (8.37), LLDPE $Mg(OH)_2$ (powder) (8.77), LLDPE $Mg(OH)_2$ (spray) (8.04), LLDPE MgO (spray) (8.49), LLDPE $Cu(OH)_2$ (spray) (8.07), and LLDPE ZnO (spray) (8.71) as shown in (**Table S3**).

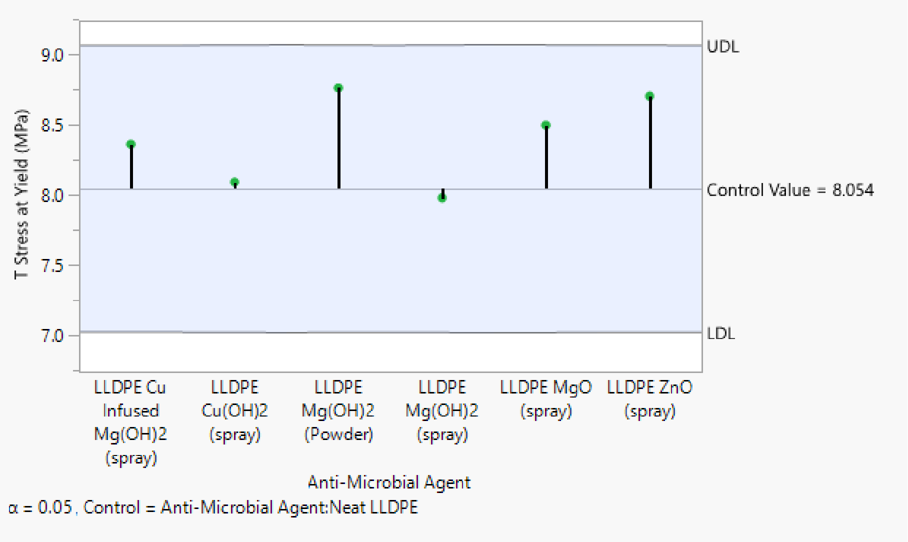

Figure S12. Comparison with control decision chart.

Table S3. Comparisons with control summary.

Anti-Microbial Agent	Lower Limit	Estimate	Upper Limit
LLDPE Cu-infused $Mg(OH)_2$ (spray)	7.031594	8.369714	9.075472
LLDPE $Cu(OH)_2$ (spray)	7.02806	8.100442	9.079006
LLDPE $Mg(OH)_2$ (Powder)	7.03176	8.77123	9.075306
LLDPE $Mg(OH)_2$ (spray)	7.027298	7.988865	9.079768

Anti-Microbial Agent	Lower Limit	Estimate	Upper Limit
LLDPE MgO (spray)	7.030039	8.50573	9.077026
LLDPE ZnO (spray)	7.031702	8.712054	9.075364

S4.9 Response to Tensile Stress at Break (MPa)

The tensile stress at break was evaluated using the following model:

$$y = \mu + \tau_r + \beta + e$$

Where y was the tensile stress at break, μ was the overall mean of tensile strength, τ_r was the treatment effect of the anti-microbial agent, β was the effect of bar weight, and e was the standard deviation.

The ANOVA for tensile stress at break indicates that after adjusting for differences in tensile bar weight, the tensile stress at break for each of the anti-microbial agents was not significantly different from the control. The p-values were as follows: LLDPE Cu-infused $Mg(OH)_2$ (spray) (P = 0.4483), LLDPE $Mg(OH)_2$ (powder) (p=0.6573), LLDPE $Mg(OH)_2$ (spray) (p=0.5376), LLDPE MgO (spray) (p=0.9428), LLDPE $Cu(OH)_2$ (spray) (p=0.4186), and LLDPE ZnO (spray) (p=0.3568) as shown in (**Table S4**).

Table S4. Parameter estimates.

Term	Estimate	Std Error	t Ratio	Prob>|t|
Intercept	26.411456	1.334561	19.79	<.0001*
Anti-Microbial Agent[LLDPE Cu-infused $Mg(OH)_2$ (spray)]	0.8242589	1.071325	0.77	0.4483
Anti-Microbial Agent[LLDPE $Cu(OH)_2$ (spray)]	-0.885026	1.07744	-0.82	0.4186
Anti-Microbial Agent[LLDPE $Mg(OH)_2$ (Powder)]	0.4805928	1.071236	0.45	0.6573
Anti-Microbial Agent[LLDPE $Mg(OH)_2$ (spray)]	-0.678435	1.086641	-0.62	0.5376
Anti-Microbial Agent[LLDPE MgO (spray)]	0.0776998	1.073674	0.07	0.9428
Anti-Microbial Agent[LLDPE ZnO (spray)]	1.0042303	1.071253	0.94	0.3568
Bar weight (mg)	-6.573736	1.27568	-5.15	<.0001*

To furtherly evaluate the anti-microbial agents, the Dunnet's method was used. The results again showed that none of the anti-microbial agents was significantly different to the control. All values fell within upper and lower decision limits as calculated via Dunnett's test. The upper limit was calculated to be between (23.572 to 23.592), and the lower limit was between (14.589 to 14.609) as shown in (**Figure S13**). All estimates fall within this range (14.589 to 23.592). The estimates for each anti-microbial agent were as follows: LLDPE Cu-infused $Mg(OH)_2$ (spray) (20.74), LLDPE $Mg(OH)_2$ (powder) (20.39), LLDPE $Mg(OH)_2$ (spray) (23.59), LLDPE MgO (spray) (23.58), LLDPE $Cu(OH)_2$ (spray) (23.59), and LLDPE ZnO (spray) (23.57) as shown in (**Table S5**).

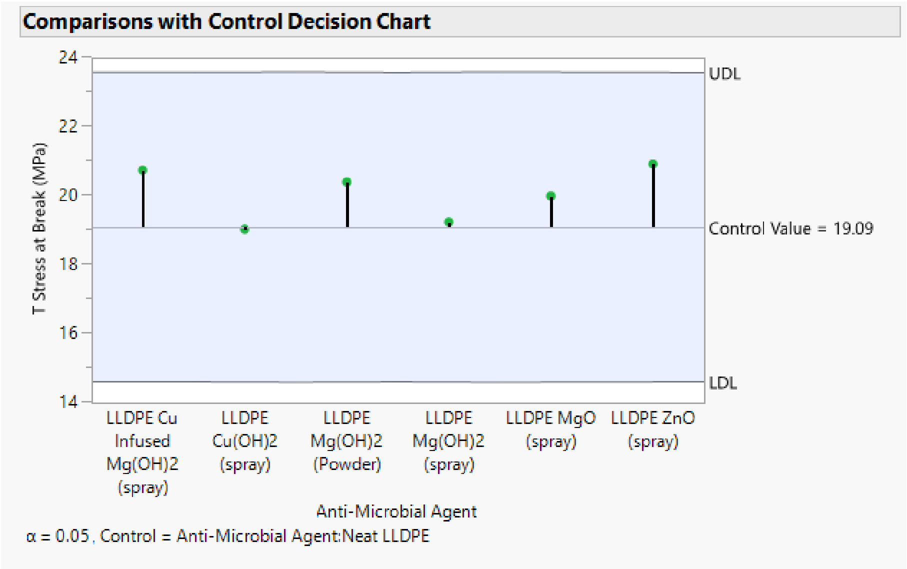

Figure S13. Comparison with control decision chart.

Table S1. Comparison with control summary.

Anti-Microbial Agent	Lower Limit	Estimate	Upper Limit
LLDPE Cu-infused $Mg(OH)_2$ (spray)	14.6083	20.73829	23.57312
LLDPE $Cu(OH)_2$ (spray)	14.5928	19.02901	23.58862
LLDPE $Mg(OH)_2$ (Powder)	14.60903	20.39462	23.57239
LLDPE $Mg(OH)_2$ (spray)	14.58946	19.2356	23.59197
LLDPE MgO (spray)	14.60148	19.99173	23.57994
LLDPE ZnO (spray)	14.60877	20.91826	23.57265

S4.10 Response to Tensile Modulus (MPa)

The ANOVA for tensile modulus indicated that after adjusting for differences in tensile bar weight, the tensile modulus for some of the anti-microbial agents, such as LLDPE $Mg(OH)_2$ (powder), LLDPE $Mg(OH)_2$ (spray), LLDPE $Cu(OH)_2$ (spray), and LLDPE ZnO (spray), were significant. However, further testing was required before a conclusion could be drawn. The p-values for various extrusion blow molded bottles were as follows: LLDPE Cu-infused $Mg(OH)_2$ (spray) (P=0.6001), LLDPE $Mg(OH)_2$ (powder) (p=0.0133), LLDPE $Mg(OH)_2$ (spray) (p=0.0338), LLDPE MgO (spray) (p=0.2332), LLDPE $Cu(OH)_2$ (spray) (p=0.0422), and LLDPE ZnO (spray) (p=0.0452) as shown in (**Table S6**).

Table S2. Parameter estimates.

Term	Estimate	Std Error	t Ratio	Prob>|t|
Intercept	174.49777	5.920302	29.47	<.0001*
Anti-Microbial Agent[LLDPE Cu-infused $Mg(OH)_2$ (spray)]	-2.521076	4.752549	-0.53	0.6001
Anti-Microbial Agent[LLDPE $Cu(OH)_2$ (spray)]	-10.19226	4.779674	-2.13	0.0422*
Anti-Microbial Agent[LLDPE $Mg(OH)_2$ (Powder)]	12.593537	4.752152	2.65	0.0133*
Anti-Microbial Agent[LLDPE $Mg(OH)_2$ (spray)]	-10.78201	4.820494	-2.24	0.0338*
Anti-Microbial Agent[LLDPE MgO (spray)]	5.8085638	4.762967	1.22	0.2332
Anti-Microbial Agent[LLDPE ZnO (spray)]	9.979132	4.752229	2.10	0.0452*
Bar weight (mg)	13.846061	5.659097	2.45	0.0212*

There is a need to do further testing using Dunnet's method. The results again showed that none of the anti-microbial agents was significantly different to the control. All values fell within upper and lower decision limits calculated using Dunnett's test. The

upper limit was calculated to be between (203.18 to 203.265), and the lower limit was between (163.329 to 163.416) as shown in (**Figure S14**). All estimates fell within this range (163.329 to 203.265). The list of estimates for each anti-microbial agent were as follows: LLDPE Cu-infused $Mg(OH)_2$ spray (185.66), LLDPE $Mg(OH)_2$ powder (200.78), LLDPE $Mg(OH)_2$ spray (177.40), LLDPE MgO spray (193.99), LLDPE $Cu(OH)_2$ spray (177.99), and LLDPE ZnO spray (198.16) as shown in (**Table S7**).

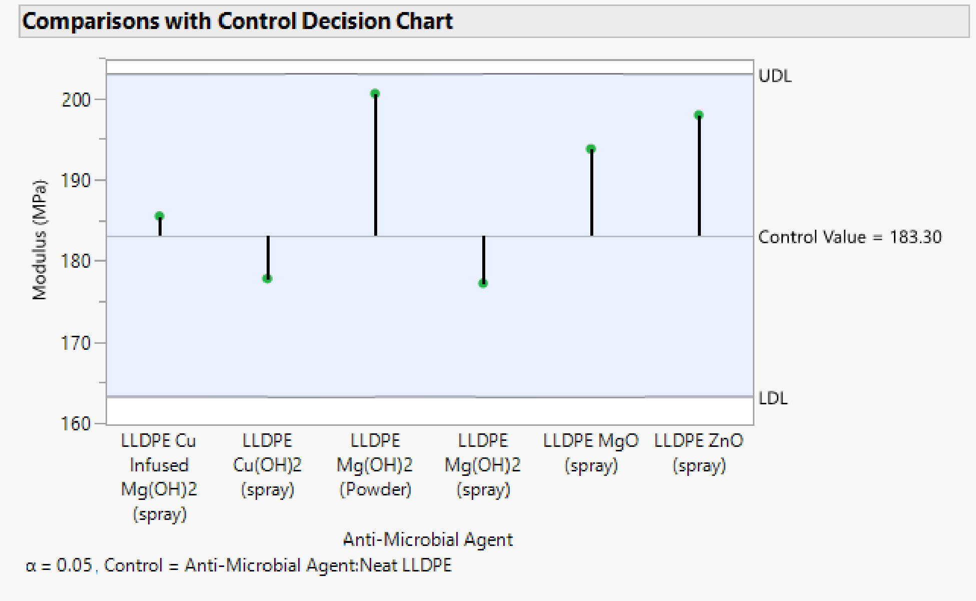

Figure S14. Comparison with control decision chart.

Table S7. Comparisons with control summary.

Anti-Microbial Agent	Lower Limit	Estimate	Upper Limit
LLDPE Cu-infused $Mg(OH)_2$ (spray)	163.4126	185.662	203.1818
LLDPE $Cu(OH)_2$ (spray)	163.3438	177.9908	203.2506
LLDPE $Mg(OH)_2$ (Powder)	163.4158	200.7766	203.1786
LLDPE $Mg(OH)_2$ (spray)	163.329	177.4011	203.2654
LLDPE MgO (spray)	163.3824	193.9917	203.2121
LLDPE ZnO (spray)	163.4147	198.1622	203.1797

S4.11 Response to Elongation at Break (%)

The ANOVA for elongation at break indicated that after adjusting for differences in the tensile bar weight, the elongation at break for each to the anti-microbial agent was not significantly different from the control. The p-values for various extrusion blow molded bottles were as follows: LLDPE Cu-infused $Mg(OH)_2$ (spray) (P=0.7893), LLDPE $Mg(OH)_2$ (powder) (p=0.4997), LLDPE $Mg(OH)_2$ (spray) (p=0.8460), LLDPE MgO (spray) (p=0.5667), LLDPE $Cu(OH)_2$ (spray) (p=0.7597), and LLDPE ZnO (spray) (p=0.4973) as shown in (**Table S8**).

Table S8. Parameter estimates.

Table .	Estimate	Std Error	t Ratio	Prob>|t|
Intercept	689.68865	16.60358	41.54	<.0001*
Anti-Microbial Agent[LLDPE Cu-infused $Mg(OH)_2$ (spray)]	3.5967069	13.3286	0.27	0.7893
Anti-Microbial Agent[LLDPE $Cu(OH)_2$ (spray)]	-4.141335	13.40467	-0.31	0.7597
Anti-Microbial Agent[LLDPE $Mg(OH)_2$ (Powder)]	-9.117698	13.32748	-0.68	0.4997
Anti-Microbial Agent[LLDPE $Mg(OH)_2$ (spray)]	2.6519041	13.51915	0.20	0.8460

Table .	Estimate	Std Error	t Ratio	Prob>|t|
Anti-Microbial Agent[LLDPE MgO (spray)]	-7.748945	13.35781	-0.58	0.5667
Anti-Microbial Agent[LLDPE ZnO (spray)]	9.1693333	13.3277	0.69	0.4973
Bar weight (mg)	11.601916	15.87102	0.73	0.4711

To further evaluate the anti-microbial agents, the Dunnet's method was used. The results again showed that none of the anti-microbial agents was significantly different to the control. All values fall within the upper and lower decision limits as calculated using Dunnett's test. The upper limit was calculated to be between (762.507 to 762.747), and the lower limit was between (650.745 to 650.988) as shown in (**Figure S15**). All estimates fall within this range (650.745 to 762.747). The estimates for each anti-microbial agent were as follows: LLDPE Cu-infused $Mg(OH)_2$ (spray) (704.75), LLDPE $Mg(OH)_2$ (powder) (692.04), LLDPE $Mg(OH)_2$ (spray) (703.81), LLDPE MgO (spray) (693.41), LLDPE $Cu(OH)_2$ (spray) (697.01), and LLDPE ZnO (spray) (710.33) as shown in (**Table S9**).

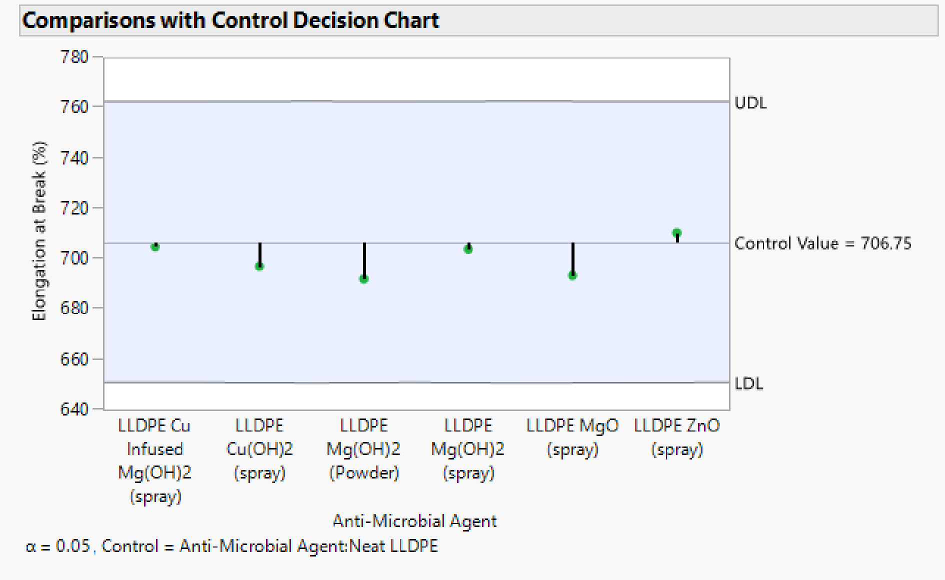

Figure S15. Comparison with control decision chart.

Table S9. Comparisons with control summary.

Anti-Microbial Agent	Lower Limit	Estimate	Upper Limit
LLDPE Cu-infused $Mg(OH)_2$ (spray)	650.9792	704.7526	762.5126
LLDPE $Cu(OH)_2$ (spray)	650.7864	697.0145	762.7054
LLDPE $Mg(OH)_2$ (Powder)	650.9883	692.0382	762.5035
LLDPE $Mg(OH)_2$ (spray)	650.7448	703.8078	762.747
LLDPE MgO (spray)	650.8944	693.4069	762.5974
LLDPE ZnO (spray)	650.9851	710.3252	762.5067

S4.12 Discussion

Considering the tensile properties as measured by tensile stress at yield, tensile stress at break, tensile modulus, and elongation at break for the n=5 sample of each condition, none of the anti-microbial agents had significant effect on the tensile properties of the thermally embossed extrusion blow molded bottles as compared to the neat control. This would indicate that any of the anti-microbial agents could be applied at the tested loading levels without effect on the blow molded bottles' tensile properties. The observed differences could become significant with further trials of larger sample sizes. A power calculation using JMP was used to determine the number of required samples to detect the stated difference in the observed measurements. A Two Independent Sample Means calculation for ant-microbial agent LLDPE $Mg(OH)_2$ (Powder) and neat LLDPE tensile modulus was

performed. In order to detect an observed difference of 12.59 with the observed standard deviations of 10.6 for the Anti-Microbial Agent $Mg(OH)_2$ (Powder) and 13.2 for the Neat LDPE n=50 samples of the control and 12 samples of the treated samples for a total sample size of n=62 were included to detect the difference with a power of 95 % and an Alpha of 5 %. Although, there are no statistically significant differences among all the anti-microbial agents, the $Mg(OH)_2$ (spray) was the closest to the neat LLDPE across all measures. This could be because the platelet shape and nano size of $Mg(OH)_2$ particles (length: 300 nm, width: 200 nm, height: 10 nm, aspect ratio: (TEM) 30±17, and (AFM) 43±25) make it very unique for thermal embossing. Further studies with larger sample sizes could show differences in tensile properties.

S4.13 Conclusion

The effect of anti-microbial agents, variation in tensile bar weight, and variation in the tensile bar thickness were statistically investigated to study their possible effects on the tensile properties of extrusion blow molded bottles. The introduction of these anti-microbial agents at this loading level (10,000 ppm and 5 sprays on each side of mold cavity) can be done without any effect on the tensile properties while providing effective anti-microbial properties to the bottles. The statistical analysis has shown that after adjusting for the variation attributed to tensile bar thickness and bar weight, none of the six types of anti-microbial agents had a significantly different effect on the control's tensile stress at yield, tensile stress at break, tensile modulus, and elongation at break.

Reference

1. Valko, K.A. Effects of extrusion blow molding internal cooling technology on HDPE container performance. M.S. Thesis, Michigan State University, East Lansing, MI, United States, June **2004**.
